# Uncovering the *Nanozostera japonica* species complex suggests cryptic speciation and underestimated seagrass diversity

**DOI:** 10.1111/nph.70355

**Published:** 2025-06-30

**Authors:** Xiaomei Zhang, Lei Yu, Yu‐Long Li, Zhaxi Suonan, Teruhisa Komatsu, Guanglong Qiu, Shaochun Xu, Shidong Yue, Min Xu, Feng Wang, Yu Zhang, Kun‐Seop Lee, Jin‐Xian Liu, Yi Zhou, Thorsten B. H. Reusch

**Affiliations:** ^1^ Key Laboratory of Marine Ecology and Environmental Sciences, Institute of Oceanology Chinese Academy of Sciences Qingdao 266071 China; ^2^ Marine Evolutionary Ecology GEOMAR Helmholtz‐Center for Ocean Research Kiel Wischhofstr. 1‐3 24148 Kiel Germany; ^3^ Laboratory for Marine Ecology and Environmental Science Qingdao Marine Science and Technology Center Qingdao 266237 China; ^4^ Field Scientific Observation and Research Station of Yellow‐Bohai Sea Temperate Seagrass Bed Ecosystems Ministry of Natural Resources Qingdao 266033 China; ^5^ Department of Biological Sciences Pusan National University Pusan 46241 Korea; ^6^ Japan Fisheries Resource Conservation Association Shintomicho Building, 3‐10‐9 Irifune, Chuo‐ku Tokyo 104‐0042 Japan; ^7^ Guangxi Key Lab of Mangrove Conservation and Utilization, Guangxi Academy of Marine Sciences (Guangxi Mangrove Research Center) Guangxi Academy of Sciences Beihai 536000 China; ^8^ Observation and Research Station of Coastal Wetland Ecosystem in Beibu Gulf Ministry of Natural Resources Beihai 536015 China; ^9^ East China Sea Fisheries Research Institute Chinese Academy of Fishery Sciences Shanghai 200090 China; ^10^ Environmental Research and Management Center Hiroshima University 1‐5‐3 Kagamiyama, Higashi‐hiroshima Hiroshima 739‐8513 Japan; ^11^ University of Chinese Academy of Sciences Beijing 100049 China

**Keywords:** chromosomal inversion, cryptic species, hybridization, *Nanozostera japonica*, seagrasses, secondary contact, triploidy

## Abstract

- The importance of marine angiosperms, or seagrasses, as foundation species for marine coastal ecosystems is in marked contrast to their low species number of only 70 described taxa. Seagrass species tend to have very similar overall morphologies dictated by hydrodynamic forces of the ocean environment. Moreover, they have inconspicuous and simple flowers, probably due to the absence of flower–pollinator interactions in the sea. We hypothesized that the seagrass species *Nanozostera japonica*, distributed from tropical to temperate latitudes in the North‐Western Pacific, contains cryptic species.
- To test this, we assembled two chromosome‐level reference genomes and conducted whole‐genome resequencing at 17 locations throughout its native distribution.
- We identified two genetically divergent clades in the north and south of its range (hereafter Nj_N and Nj_S) that diverged some 4.16 million years ago. Hybrid individuals at a contact zone were either diploid F1‐crosses or featured triploidy, indicating reproductive isolation. A large (42 Mb) reciprocally fixed chromosomal inversion between Nj_N and Nj_S possibly contributed to the formation of the reproductive isolation.
- We conclude that the *N. japonica* species complex is actually consisting of two different species. Further population genomic studies may reveal additional cryptic species, solving the conundrum why there are so few described seagrass species today.

## 摘要

- 作为沿海生态系统的基石物种，海洋被子植物海草的重要性与其极低的物种数量形成鲜明对比——目前全球仅记载海草70余种。受海洋水动力环境的塑造作用，海草物种整体呈现出高度相似的形态特征。此外，海草花器官结构简单而不起眼，这很可能源于海洋环境中缺乏花‐传粉者互动机制。因此，我们推测纵跨温带至热带的西北太平洋海草物种——日本鳗草(*Nanozostera japonica*)，可能包含隐存种。
- 为了检验这一猜想，我们组装了两个具有代表性的染色体水平的参考基因组，并对其源生地分布范围的17个地理群体进行了全基因组重测序。
- 研究结果显示日本鳗草显著分化为南北两个遗传分支，分化时间为4.16百万年前；两个遗传分支的接触区内发现少量杂交个体，且仅呈现两种遗传状态——第一代杂交后代(F1)或三倍体，这表明两个遗传分支存在生殖隔离。同时，发现两个遗传分支之间存在一个大型染色体转置(42 Mb)的纯合差异，可能对生殖隔离的形成发挥作用。
- 我们认为目前定义的日本鳗草(*N. japonica*)实际上包含两个不同的物种，未来进一步加强海草群体基因组学研究，可能会发现更多的隐存种，从而解答海草物种多样性极低的谜题。

## Introduction

Cryptic species, different taxa hidden under any contemporary species description due to morphological similarity, are widespread in nature (Bickford *et al*., 2007) and may represent a substantial but understudied part of species‐level biodiversity (Fiser *et al*., 2018). In the past decades, substantial cryptic diversity has been uncovered in animals, but only a small proportion in plants (Bickford *et al*., 2007; Shneyer & Kotseruba, 2015; Struck *et al*., 2018). In the latter group, the presence of cryptic species is a particular problem when plants display few distinguishable morphological traits (e.g. mosses, McDaniel & Shaw, 2003), highly plastic morphological variation, or frequent hybridization and polyploidization (e.g. ferns, Kinosian *et al*., 2020; Wei *et al*., 2021).

The ocean represents a particularly dominant environment in terms of trait evolution. Strong currents, wave action, and the need for hydrophilous pollination impose strong selection for a convergent morphology and physiology for the unique group of marine angiosperms or seagrasses, a polyphyletic group of flowering plants that returned to the marine realm *c*. 100 million years ago (Ma) (Unsworth *et al*., 2022) and that are distributed along shallow coastlines of all continents except Antarctica (Short *et al*., 2007). Ocean hydrodynamic forces including currents (unidirectional) and waves (oscillating) (Les *et al*., 1997; Olsen *et al*., 2016) resulted in the convergent evolution strap‐like flexible leaves emerging from a basal meristem, anchored in the sediment by horizontal rhizomes and roots (Moreira‐Saporiti *et al*., 2023). Probably owing to the lack of pollinator‐flower coevolution, seagrass flowers have also been simplified and lack a corolla, while male flower usually feature filiform pollens in combination with bifurcated stigmata in female flowers (Kuo & Den Hartog, 2001).

Despite their global distribution and enormous ecological importance in supporting highly productive ecosystems with abundant biodiversity and important ecosystem functions, their described species number is low (70 species or so, Les *et al*., 1997; den Hartog & Kuo, 2006). This is even more striking when comparing numbers against terrestrial vascular plants (350 000–400 000 species, Walker, 2013). Moreover, seagrass phenotypes are highly plastic within species (Pazzaglia *et al*., 2021), which further complicates species‐level determination. Hence, we hypothesize here that there are cryptic seagrass species, which are defined to show (1) separate genome‐level monophyletic genetic clades (i.e. phylogenetic species concept), (2) a corresponding genetic distance comparable to other described seagrass species (i.e. evolutionary species concept), and (3) reproductive isolation (i.e. biological species concept) (Paris *et al*., 1989; De Queiroz, 2007).

Our focal species, *Nanozostera* (previously *Zostera*) *japonica*, is an intertidal dwarf eelgrass with a native distribution range in the North‐Western Pacific, while it was introduced to the Pacific coastlines of North America from Japan through oyster shipments in the early 20^th^ century (Harrison & Bigley, 1982). It is widely distributed from southern Vietnam (at *c*. 10°N) to Sakhalin, Russia (at *c*. 55°N) in the North‐Western Pacific (Shin & Choi, 1998), representing one of the few seagrass species that occur from temperate to tropical areas (den Hartog, 1970). *Nanozostera japonica* and its sister species, the European dwarf eelgrass *Nanozostera noltii*, possess highly similar and plastic morphological characters in vegetative and reproductive structure, with the key taxonomic feature for identification being tiny differences on the blade apex (Kuo & Den Hartog, 2001). To complicate matters, *N. japonica* exhibits highly variable phenotypes among different geographic environments, such as peak biomass, growth form (annual or perennial), reproductive phenology, reproductive ratio (asexual vs sexual reproductive investment), and seed set, especially between southern and northern populations in mainland China (e.g. Suonan *et al*., 2017; Yue *et al*., 2020; Zhang *et al*., 2020; Ito *et al*., 2021). In addition, a previous microsatellite study based on 24 loci throughout the distribution range also revealed pronounced genetic differentiation between northern and southern populations (Zhang *et al*., 2024), but was unable to quantify or date this divergence relative to other delineated seagrass species.

Here, we hypothesized that contemporary *N. japonica* populations actually consist of multiple species, rather than one species displaying extreme phenotypic plasticity and ecotypic differentiation across a thermal range of *c*. 6–29°C of mean annual ocean temperature. To test this notion, we used genome‐wide sequence data throughout the distribution range of *N. japonica* at multiple locations, while comparing the genomic divergence to its neighboring noncryptic taxa *N. noltii* using the multispecies coalescent (MSC, Bryant *et al*., 2012). By calibrating the phylogenetic tree through the available divergence time between *N. japonica* and *Zostera marina* in line with previous work (Yu *et al*., 2023), we were able to estimate divergence times, further supporting any conclusions regarding the species status.

## Materials and Methods

Further detailed description of the methods used in this study can be found in Supporting Information Methods S1.

### Seagrass *Nanozostera japonica*

Occurring in its native range in the North‐West Pacific, *Nanozostera japonica* (Asch. & Graebn.) Toml. & Posl. is one of the few seagrass species distributed across both tropical and temperate coastlines, displaying highly plastic morphological traits and life history strategies among different geographical locations. Thus, it provides a good model to examine the potential cryptic speciation under such a wide range of environmental gradients. We adopted the genus status of *Nanozostera* proposed repeatedly in recent taxonomic work (Tomlinson & Posluzny, 2001; Sullivan & Short, 2023) as one of four genera within the family Zosteraceae based on molecular markers and molecular genetic distances (Coyer *et al*., 2013).

### Genome assembly and annotation

We assembled a chromosome‐level reference genome for a sample collected from Huiquan Bay, Qingdao, northern China (36°03′05″N, 120°20′17″E), as the reference for all further population genomic analyses. After obtaining the results from the population genomic results showing a deep divergence between southern and northern sites, we decided to assemble an independent southern reference genome which also served to verify the genomic inversion. The second chromosome‐level reference genome was based on a sample collected from Pearl Bay, Fangchenggang, southern China (21°35′52″N, 108°12′46″E). Genes were predicted using the evidence from both *ab initio* gene predictors and protein and transcript alignments.

### Large‐scale sample collection, resequencing, and genetic population structure

Samples were collected from the native range of *N. japonica*. The sampling design aimed to evenly cover the native distribution range with the exception of Russia. Seventeen *Nanozostera noltii* samples were collected from Germany (54°40′57″N, 9°59′47″E) by snorkeling. Two seedlings of *Zostera marina* collected from the field (in Germany, Baltic Sea, 54°23′33.7″N; 10°11′34.1″E), were cultured in the lab until they were large enough for DNA extraction. We conducted whole‐genome resequencing for 307 *N. japonica* samples from 17 populations throughout its biogeographic range in the North‐Western Pacific, 17 *N. noltii* samples from Germany, and two *Z. marina* seedlings cultured in the lab. Gatk4 (Van der Auwera *et al*., 2013) was used to conduct joint SNP calling. Clone detection was done based on both shared heterozygosity and the number of fixed differences. For each genet, only the ramet with the lowest data missing rate was kept, while all redundant ramets were removed. PCA, neighbor‐joining tree, and Structure v.2.3.4 analysis (Pritchard *et al*., 2000) were used to analyze the genetic population structure. The clean next‐generation sequencing (NGS) reads were mapped to the chloroplast reference genome of *N. japonica* (Chen *et al*., 2021) to build a chloroplast haplotype network.

### Detecting triploidy using variant read frequency histograms

Homologous chromosomes that carry bi‐allelic heterozygous loci would display an equal number of reference alleles (i.e. REF allele in the vcf file) and variant alternate alleles (ALT allele in the vcf file) in diploid individuals, corresponding to a variant allele frequency of 0.5. This will lead to a peak at 0.5 in the variant read frequency (VRF) histogram (VRF, the number of NGS reads supporting the ALT allele/total number of NGS reads). Similarly, triploid individuals may either display 0, 1, 2, or 3 copies of the variant allele, corresponding to a variant allele frequency of 0, 1/3, 2/3, and 1, respectively, producing VRF peaks at 1/3 or 2/3 or both. For each sample, all the heterozygous genotypes were selected, the VRF was calculated, and a histogram was plotted.

### Detecting F1 hybrids and higher‐order hybridization

F1 hybrids, the direct offspring of two different species or highly diverged clades, will consistently lead to heterozygous genotypes as a result of many fixed genomic differences. Higher‐order hybrids (F2 or backcrosses), in turn, will lose 50% of that heterozygosity through Mendelian segregation. Hence, we first identified the fixed differences between Nj_N and Nj_S, and then checked the genotypes of hybrids at those single nucleotide polymorphisms (SNPs). For each hybrid sample, the proportion of heterozygous genotypes was calculated, that is the number of heterozygous SNPs/total number of SNPs with available genotypes. A value equal to or very close to 100% would indicate F1 hybrids.

A triangle plot for hybrid index and interclass heterozygosity was plotted using the R package triangular (Wiens *et al*., 2025) based on the SNPs with fixed differences between Nj_N and Nj_S. To save running time, the input dataset was thinned to 58 029 SNPs by keeping only one SNP within a 3000‐bp window.

### Time‐calibrated phylogenetic tree

Two *Z. marina* seedlings (sampled in Germany, Baltic Sea, site Falckenstein at 54°23′33.7″N; 10°11′34.1″E) and all unique genets for *N. japonica* and *N. noltii* were subjected to a joint SNP calling with subsequent filtering steps (details see full Materials and Methods section). A MSC analysis implemented in Snapp was conducted to build a time‐calibrated phylogenetic tree, using the divergence time between *Z. marina* and *N. japonica* as calibration point (Yu *et al*., 2023).

### Karyotyping

As karyotyping requires live plants, first we used microsatellite markers to distinguish putatively diploid and triploid live plants based on the DNA extracted from a small piece of leaf of newly collected *N. japonica* plants from the population YUL with putative triploidy (Datasets S1, S2). In a subsequent step, these same live plants were subjected to flow cytometry to determine the karyotypes.

### Comparison of the two chromosome‐level reference genomes

To check whether inversions might play a role in hybrid sterility, we compared the Nj_N and Nj_S reference genomes based on both synteny comparison and sequence alignment. To reveal which samples were homozygous or heterozygous for the inversion, a PCA was conducted based on the 131 306 SNPs located in the inversion region.

## Results

### Genome assembly

A chromosome‐level reference genome for *N. japonica* (HQ) was constructed using a combination of short sequence reads, PacBio long reads, and HiC reads (Fig. S1). Similar to the model seagrass species *Z. marina*, it also contained six pairs of chromosomes (Fig. S1). The final assembly with contig N50 of 52.29 Mb (size of the six main chromosomes: 966.49 Mb; Table S1) and Busco score of 95% (Fig. S2) demonstrated a high level of completeness of the genome compared to other sequenced seagrass genomes (Ma *et al*., 2024). Although the size of the six main chromosomes was > 4 times as large as that for *Z. marina* (966.49 Mb vs 219.29 Mb, excluding unanchored sequences), the number of protein coding genes (22 074; Table S2) was similar to estimates for *Z. marina* (21 483; Ma *et al*., 2021), and 93% of those could be annotated by public databases (Pfam, InterPro, EggNOG, and Uniprot). Further details are given in Table S2.

### Whole‐genome resequencing and genet detection

Upon mapping whole‐genome resequencing data to the reference genome, we obtained 2781 296 high‐quality SNPs for 304 samples (Table S3). Clone mates in each of the *N. japonica* populations were detected based on both shared heterozygosity and the number of fixed genetic differences. Subsequently, redundant ramets of the same genet were excluded for the population‐level analyses (details see Methods S1). We detected 19 genets with ≥ 2 ramets, and the number of ramets for each clone ranged from 2 to 16 (Fig. S3; Table S4). The largest clones were found in South Korea (population YUL) and in Japan (population NAN), with 16 and 8 ramets, respectively.

After excluding the redundant ramets of the same genet, we obtained 2715 951 SNPs for 258 unique genets (hereafter referred to as ‘Core Dataset’, size of the reference genome: 966.49 Mb). The resequencing data were also mapped against the chloroplast reference genome (genome size: 146 090 bp), and we obtained 427 high‐quality SNPs without any missing data among the same 258 samples as in the ‘Core Dataset’ (hereafter referred to as ‘cpDataset’).

### Global genetic population structure

**Fig. 1:**
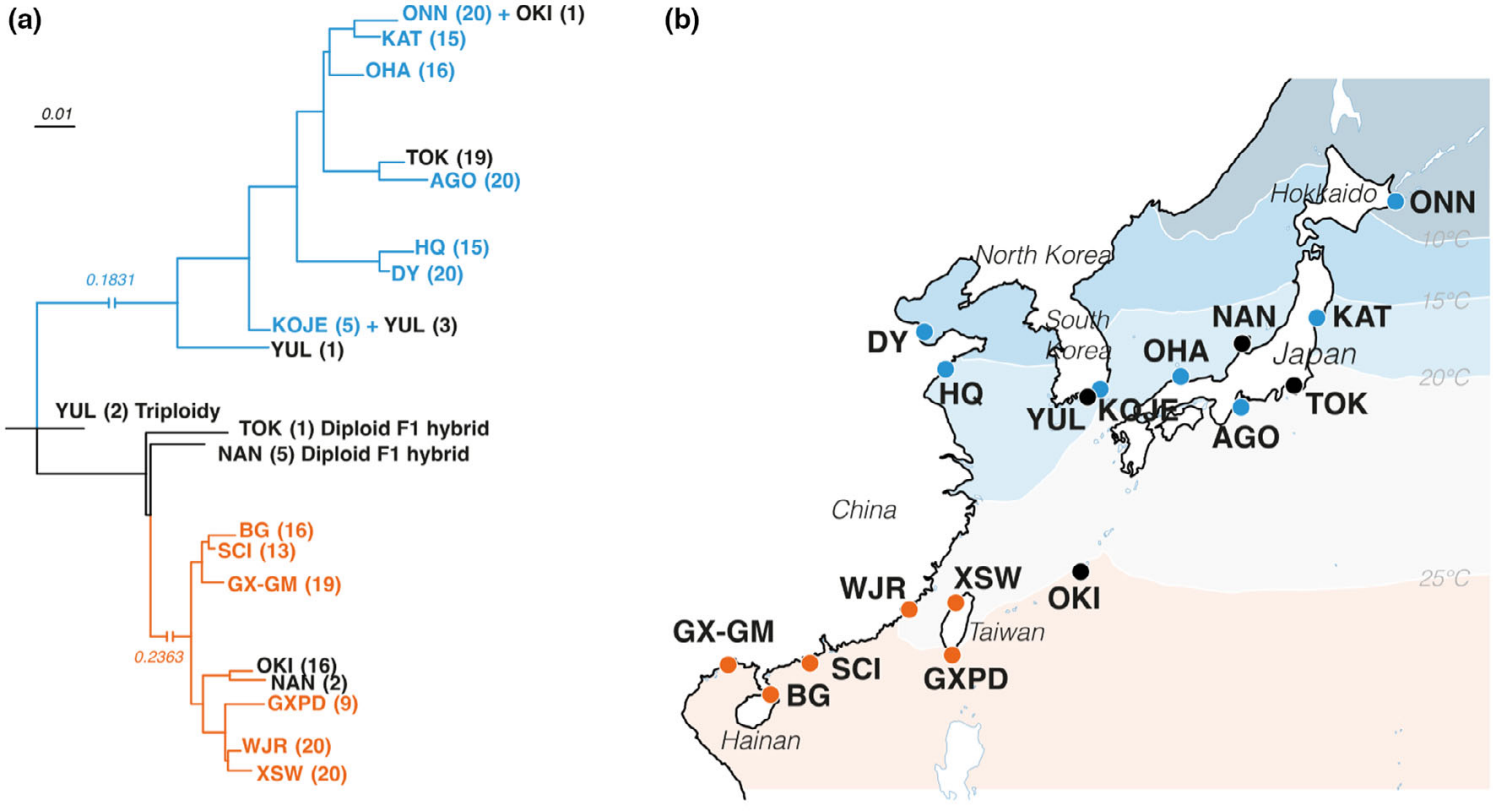
The *Nanozostera japonica* species complex consists of two deeply divergent genetic clades. (a) Neighbour‐Joining (NJ) tree is constructed using pairwise genetic distances (1‐ibs) calculated based on the Core Dataset, which divides the samples into three groups. The two groups dominating the southern and northern distribution range are denoted ‘Nj_S’ and ‘Nj_N’, respectively. AGO (Ago Bay), BG (Beigang Island), DY (Dongying), GX‐GM (Guiming village, Guangxi Province), GXPD (Pingdong, Gaoxiong), HQ (Huiquan Bay), KAT (Katsura‐jima Island), KOJE (Koje Island), NAN (Nanao Bay), OHA (Ohashi River), OKI (Okinawa Island), ONN (Onneto Lake), SCI (Shangchuandao Island), TOK (Tokyo Bay), WJR (Wujiang River), XSW (Xiangshan Wetland), YUL (Yulpo). Label colors correspond to the color used for different populations in (b). The two values ‘0.1831’ and ‘0.2363’ indicate the genetic distances. (b) Sampling locations in the North‐West Pacific with sea temperature isotherms. Locations occupied exclusively by Nj_S or Nj_N are marked with orange or blue colors, respectively. All other populations are marked in black. Those populations either show hybrids (F1 or triploidy) or co‐existence of both clades, which is likely caused by migrants via ocean currents, human activities, or bird‐mediated dispersal.

**Fig. 2:**
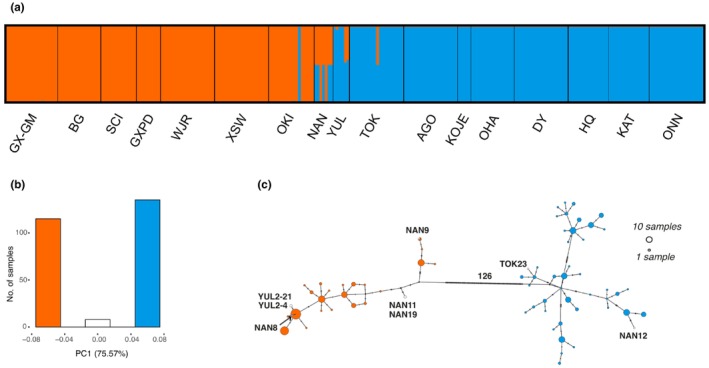
Genetic population structure of the *Nanozostera japonica* species complex. (a) Structure plot for *K* = 2 based on 13 056 single nucleotide polymorphisms (SNPs) randomly selected from the Core Dataset with minimum pairwise distance > 5 kb. All 20 independent repeats support the same pattern. The southern (Nj_S) and northern group (Nj_N) represent two unique genetic components in the Structure analysis, with an intermediate group showing admixture of the two genetic components. (b) PCA based on the Core Dataset. PC1 explains 75.57% of the variance, based on which all samples are divided into the same three groups shown in (a). (c) Chloroplast haplotype network based on the full chloroplast genomes (146 090 bp, 427 SNPs). Nj_S and Nj_N differ by 126 mutational steps. Eight genets comprising an intermediate group are found in both clusters (only labeled haplotypes in ‘c’).

A Neighbour‐Joining tree (Fig. 1) based on the Core Dataset revealed a pronounced population structure into two distinct genetic groups with some intermediate populations that were also supported by the Structure analysis and the PCA (PC1, 75.57% of variance explained, Fig. 2a,b). Two deeply divergent clades showed south–north allopatric distribution (named as ‘Nj_S’ and ‘Nj_N’, respectively), while the third intermediate group involved 1, 2, and 5 genets of the populations TOK, YUL, and NAN, respectively. A Structure analysis found the strongest support for *K* = 2 clusters (13 056 SNPs; 20 independent repeats) and identified Nj_S and Nj_N as two unique genetic components (Fig. 2a), while the third group showed admixture of Nj_S and Nj_N, supporting an intermediate status.

The chloroplast haplotypes supported the strong divergence of nuclear genomes (Fig. 2c). Such an analysis is in principle not able to resolve intermediate individuals because chloroplast haplotypes are inherited maternally. Accordingly, in the chloroplast haplotype network based on the cpDataset (Fig. 2c), there were only two major clusters separated by 126 mutational steps, while 8 genets belonging to the intermediate group based on nuclear SNPs were found in either cluster, depending on the origin of the maternal individual of that sample.

According to the nuclear population structure (Figs 1a, 2a,b), only Nj_S individuals were among six locations in the southern distribution range (depicted orange in Fig. 1b), whereas seven locations in the northern distribution range consisted entirely of Nj_N plants (blue, Fig. 1b). In the other four geographically intermediate populations, genets from the admixed group were found in three of them (YUL, NAN, and TOK), whereas OKI showed coexistence of both Nj_S and Nj_N (16 Nj_S + 1 Nj_N).

### Evidence for genomic divergence supporting species status of Nj_S and Nj_N

**Fig. 3:**
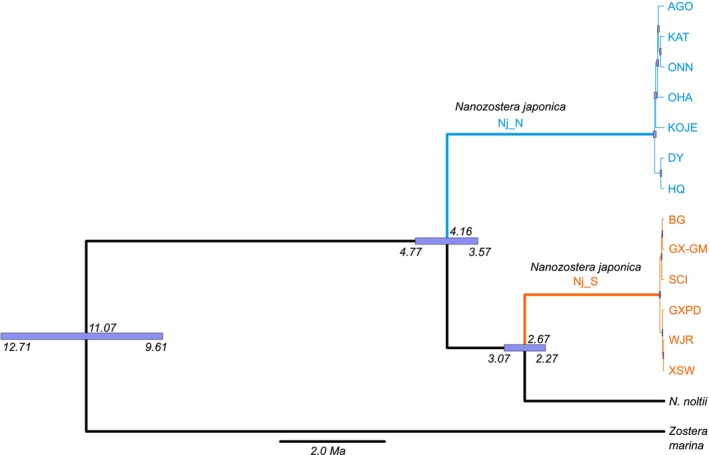
Time‐calibrated phylogenetic tree of members of the Zosteraceae using the multispecies coalescent. Joint SNP calling and filtering were conducted for *Nanozostera japonica*, *Nanozostera noltii*, and *Zostera marina*. For *N. japonica*, only non‐admixed locations marked with orange or blue colours in Fig. 1(a) are included. Two unique genets were used to represent each population. As for *N. noltii* and *Z. marina*, three and two genets were used, respectively. The same analysis was repeated three times with different sample sets, resulting in same topology and similar estimates. The Snapp analysis was run three times independently based on 10 454 randomly selected SNPs with a minimum pairwise distance > 5 kb. The time calibration is conducted based on the calibration point for *N. japonica* and *Z. marina*, where the divergence time is available (Yu *et al*., 2023). The horizontal bars spanning the nodes indicate 95% highest posterior density intervals.

Next, we estimated the divergence time among both clades Nj_S and Nj_N, and the sister species *N. noltii*, using the MSC implemented in Snapp (Bryant *et al*., 2012). As any admixture biases the Snapp analysis, which requires a bifurcating history of population‐level divergence, we focused on *N. japonica* populations belonging to either identified monophyletic clade (orange or blue colors in Fig. 1b). Nj_S was more closely related to *N. noltii* than to Nj_N (Fig. 3). Nj_S and *N. noltii* diverged at 2.67 Ma (95% highest posterior density (HPD): 3.07–2.27 Ma), sharing the common ancestor with Nj_N at 4.16 Ma (95% HPD: 4.77–3.57 Ma). Since the species status of *N. noltii* has been well accepted, both Nj_S and Nj_N should be considered separate species as well as they feature a deeper divergence time.

### Hybridization upon secondary contact and detection of F1 hybrids

Some genotypes/genets were intermediate to individuals in Nj_S and Nj_N and showed genetic admixture based on a Structure analyses (Fig. 2a), suggesting that they represented hybrid individuals emerging upon secondary contact. We also introduced another index based on the fixed SNPs between Nj_S and Nj_N to detect F1 hybridization (details see Methods S1). For the 912 092 SNPs showing fixed differences between Nj_S and Nj_N in the Core Dataset, six out of eight hybrid samples were heterozygous at > 99% of the loci (Table 1), indicating that they were first generation of hybrids between Nj_S and Nj_N (i.e. F1 hybrids), as any subsequent sexual events following F1 would significantly reduce the proportion of heterozygous loci. The F1 status of those six hybrids was also supported by a triangle plot for hybrid index and interclass heterozygosity (Fig. S4). Also, those six hybrid samples consisted of almost equal amount of Nj_S and Nj_N components in the Structure analyses (Fig. S5). The hybrid status of the third group was also supported by the chloroplast haplotype network (Fig. 2c).

**Table 1:** Genomic heterozygosity for eight hybrid genets of the *Nanozostera japonica* complex identified at three sampling locations, based on a total of 912 092 single nucleotide polymorphisms (SNPs) that displayed fixed differences between Nj_S and Nj_N.

Population	Genet ID	*n*_All_	*n*_He_	*F*_He_
NAN Nanao bay, Japan	NAN11	905 228	898 702	0.9928
	NAN12	906 356	899 749	0.9927
	NAN19	908 345	903 427	0.9946
	NAN8	683 800	679 128	0.9932
	NAN9	886 757	880 904	0.9934
TOK Tokyo bay, Japan	TOK23	892 648	887 712	0.9945
YUL Yulpo, Koje Bay, South Korea	YUL2‐21	903 635	847 528	0.9379
	YUL2‐4	896 152	775 251	0.8651

*F*_He_, *n*_He_/*n*_All_; *n*_All_, the number of loci with a reliable genotype call for the target sample; *n*_He_, the number of observed heterozygous loci in the individual sample.

### Existence of triploid hybrids points to reproductive isolation

**Fig. 4:**
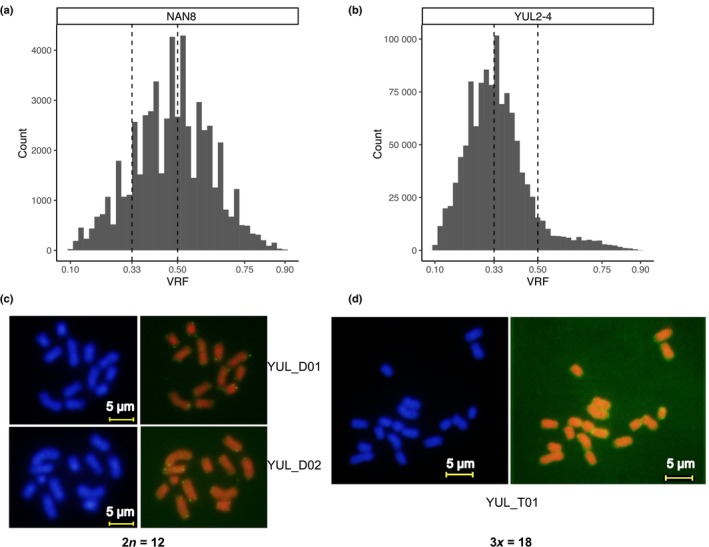
Co‐existence of diploidy and triploidy within a population of *Nanozostera japonica*. (a, b) Variant read frequency (VRF) diagrams to support the ploidy status of individuals, based on the proportions of next‐generation sequencing reads supporting the variant allele at all heterozygous single nucleotide polymorphism loci. (a) For diploid individuals, the numbers of reference (REF) and alternate (ALT) alleles are equal as in the sample (NAN8) that displays a VRF of 0.5 for both alleles. (b) The VRF histogram of samples YUL2‐4 support a peak at 0.33 indicating that the ALT allele is present in one of the three homologous chromosomes. (c, d) Karyotyping to check the number of chromosomes. (c) Karyotyping for two diploid samples YUL_D01 and YUL_D02 displaying 12 chromosomes. (d) Karyotyping for a triploid sample YUL_T01 displaying 18 chromosomes.

In some of the genetically admixed individuals, we suspected the existence of polyploidy (Dufresne & Hebert, 1994). To test this, we plotted the histogram of VRF for each of the hybrid samples based on all the bi‐allelic heterozygous loci (see Methods S1). Under diploidy, we expected two different alleles with roughly equal read counts at those loci, corresponding to a peak at VRF = 0.5 (Fig. 4a). In two genets from a Korean population, YUL, we also found a different pattern (Fig. 4b) with a peak at VRF = 0.33, suggesting, as one possible scenario, a triploid status with two sets of reference homologous chromosomes and one set of variant homologous chromosomes. We then verified the inferred ploidy level by karyotyping. Nine microsatellite markers were selected to detect putatively triploid individuals based on the genotyping results at 19 loci for 19 triploid individuals (Dataset S1). Four putatively diploid and 119 putatively triploid individuals were recovered based on the segregation of three alleles at nine microsatellite markers, respectively, demonstrating the dominance of triploids at that site (Methods S1; Dataset S2). Subsequent karyotyping of three individuals confirmed the ploidy levels inferred from microsatellite markers (Fig. 4c,d).

**Fig. 5:**
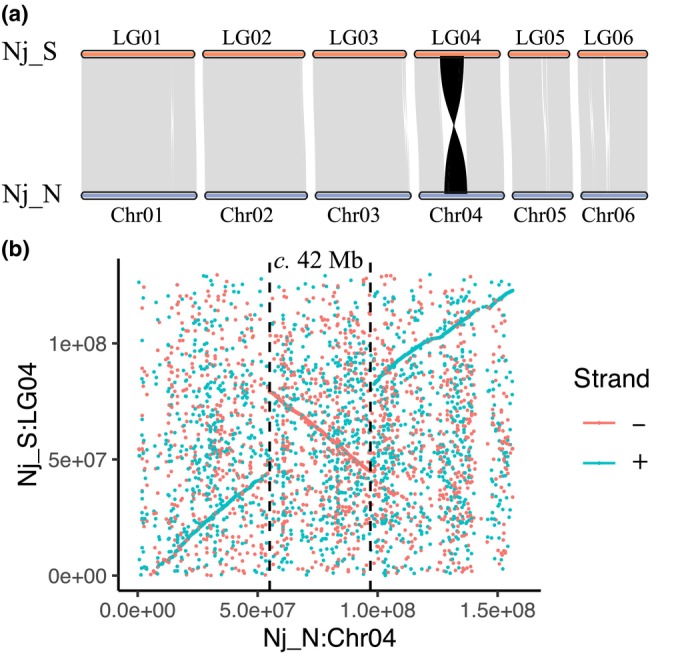
A large chromosomal inversion (*c*. 42 Mb) between two putative *Nanozostera japonica* species Nj_S and Nj_N. (a) Pairwise synteny comparison between Nj_S and Nj_N based on coding genes, with matches between two genomes connected by lines. (b) DNA sequence alignment. All the maximal unique matches of 20 bp between the two input sequences are identified, which are further clustered into closely grouped sets. All clusters with a length of > 1000 bp are shown without any filtering. The region between the two vertical dashed lines represents a large reversion of *c*. 42 Mb in size.

To further identify the genetic basis for the reproductive barrier between Nj_N and Nj_S, we *de novo* assembled a chromosome‐level reference genome for the southern clade Nj_S (size of six main chromosomes: 761.42 Mb, Figs S2, S6; Tables S1, S2). The two genomes of *N. japonica* showed a large inversion with a size of *c*. 42 Mb at chromosome 4 (Chr04 of Nj_N vs LG04 of Nj_S; Fig. 5). A PCA based on the 131 306 SNPs located in the inversion region (Nj_N, Chr04: 55648726–96617151) demonstrated that all individuals assigned to Nj_N and Nj_S based on the SNP pattern were fixed for different inversion states, whereas the hybrids (F1 hybrids + triploid hybrids) showed heterozygosity for both inversion types (Fig. S7). Thus, one plausible scenario for the emergence of triploidy is unreduced diploid gametes of F1 individuals merged with a normal Nj_N haploid gamete, resulting in a combination of heterogeneous sets of haploid chromosomes from Nj_S and Nj_N, respectively.

## Discussion

### Cryptic speciation within the *N. japonica* species complex

With the increasing availability of DNA sequence markers or population‐level genome sequences, cryptic species have been reported in an increasing number of vascular plants (e.g. Grundt *et al*., 2006; Okuyama & Kato, 2009; Abdelaziz *et al*., 2011; Esteves & Vicentini, 2013; Michalski & Durka, 2015; Ji *et al*., 2020; Boucher *et al*., 2021; Ruiz *et al*., 2021; Tian *et al*., 2023). The key tenet of our study was that a plant group with a convergent morphology and physiology dictated by the dominant ocean environment, marine angiosperms or seagrasses, feature more species than were previously discovered by morphology‐based taxonomic methods (Figs S8, S9; Table S5). Since most of the morphological characteristics show higher variation within than between presumed species, it is difficult to distinguish between the two species via classic taxonomic methods (Figs S8, S9). As a case in point, we found that in two divergent groups of *N. japonica*, coalescent based phylogenetic reconstruction points to a divergence between a southern (Nj_S) and a northern (Nj_N) clade that dates back to the early Pliocene, some 4.16 Ma (95% HPD: 4.77–3.57 Ma) ago. This is the first delimitation of cryptic species in any seagrass species through whole‐genomic data.

Based on our population genomic analyses, we also found evidence for reproductive barriers between the two cryptic species, as their hybrids were either (partial) sterile triploid individuals or diploid F1 crosses, while no higher‐order crosses were detected. The only two triploid genets were found in South Korea (YUL), and they dominated that site with 76% (19 out of 25) of the samples.

Population YUL is cooccupied by both pure Nj_N (*n* = 4) and triploid genets (*n* = 2). Based on shared heterozygosity index, the triploid samples were assigned to two unique genets, with a threshold setting to 0.9. Since such high level of divergence is already similar to that between sexually generated individuals, they are more likely to be different genets. F1 hybrids might encounter segregation problems during meiosis and will sometimes form diploid gametes. The subsequent fusion of diploid and normal haploid gametes will then lead to sterile triploidy. This process might happen in any F1 hybrids, and thus different triploid genets can be formed independently.

One of the triploid genets consisting of 16 ramets was estimated to occupy *c*. 900 m^2^, currently representing the largest genet of *N. japonica* ever reported. The second largest genet was found in the population NAN, which was a first‐generation (F1) hybrid. Our results indicate that hybrids might be favored by heterosis effects and thus may reveal higher fitness than nonhybrid individuals.

The divergence in some phenotypic traits also support species status for Nj_S and Nj_N. For example, most subtropical to tropical *N. japonica* belonging to Nj_S populations flower early in spring or even twice in a year with very low flowering frequency or seed set, while the temperate ones generally flower once in a year during summer to autumn with high sexual reproductive effort (Nakaoka & Aioi, 2001; Zhang *et al*., 2020; Qiu, 2021).

The divergence between Nj_N and Nj_S is consistent with other reported intraspecific genetic splits and (cryptic) speciation events arising along the North‐Western Pacific coastline driven by geographical isolation between different marginal seas due to sea‐level changes in the Miocene to middle Pliocene (Ni *et al*., 2014). Recent studies propose that local adaptation subsequent to glacial isolation may contribute to intraspecific variations in marine species organisms within this area (e.g. Zhao *et al*., 2017; Takeuchi *et al*., 2020; Lee *et al*., 2021). Nevertheless, taking into account the intermittent reconnection of marginal refugia during postglaciation periods since 4.16 Ma, it is conceivable that local adaptation to distinct environments (e.g. temperature) has played an additional role for driving the genetic divergence within the *N. japonica* species complex.

### Reproductive isolation among two cryptic seagrass species

Natural hybridization (Arnold, 1997) and polyploidization (Wood *et al*., 2009) are widespread among angiosperms. The hybrids detected in this study were located in an area significantly affected by either the warm Kuroshio Current or its derivative, the warm Tsushima Current. Both are powerful northward currents and have probably led to a transport of seagrass leaf shoots from the southern clade. Using an index based on fixed genetic differences between putative species (Table 1) was a powerful tool in accurately distinguishing F1 hybrids from higher‐order hybrids in *N. japonica* samples with indistinguishable plant morphology. In addition, VRF histograms were informative to distinguish triploidy from diploidy. Triploid individuals are often rendered sterile or semi‐sterile due to meiotic irregularities and high frequency of aneuploid gametes (Ramsey & Schemske, 1998; Ramsey & Ramsey, 2014), consistent with our finding that hybridization between Nj_S and Nj_N likely stops at a triploid state. Allo‐triploids may appear in the F2 generation originating from backcrossing or self‐fertilization among interspecific F1 hybrids (Ramsey & Schemske, 1998). Such interspecific F1 hybrids are more likely to form unreduced gametes than either of the parental species (Ramsey & Schemske, 1998; Zhang *et al*., 2010). The subsequent fusion of an unreduced gamete with a normal reduced gamete will then lead to triploid formation (Bretagnolle & Thompson, 1995; Ramsey & Schemske, 1998; Moghe & Shiu, 2014), consistent with our findings.

Based on a *de novo* genome assembly of Nj_S, we identified a large‐scale chromosomal inversion (*c*. 42 Mb) distinguishing both clades (Figs 5, S7). Inversions are known to suppress recombination in heterozygotes (Wellenreuther & Bernatchez, 2018; Huang & Rieseberg, 2020), disturb the normal meiosis process and produce unbalanced gametes (Torgasheva & Borodin, 2010; Termolino *et al*., 2019), contributing to reproductive isolation between hybridizing taxa (Hoffmann & Rieseberg, 2008; Lowry & Willis, 2010; Hooper *et al*., 2019). However, it will be possible for genetic exchange in other parts of the genome (Le Moan *et al*., 2024). Thus, it is unlikely that the genomic inversion is directly responsible for the two species of *N. japonica* to become reproductively isolated. However, the inversion may contain loci involved in local adaptation and prevent recombination between alleles that collectively support local adaptation (e.g. flowering time), which in turn can promote genetic isolation. After the reproductive isolation is formed, the two species will further diverge, and eventually normal meiosis process in the F1 hybrids might be in disorder, leading to the formation of diploid gametes.

Along the same lines, it is probably not a coincidence that the largest genet reported in this study (covering at least 900 m^2^) was triploid, given that this ploidy level most often represents a dead ending for further sexual reproduction (see above), leaving only propagation via vegetative reproduction, as is the case in other recently discovered cases (Karpavičienė, 2017; Kutlunina *et al*., 2017). The identified genet size of 900 m^2^ is modest compared with other reported seagrass genets extending over tens to hundreds of km (e.g. Arnaud‐Haond *et al*., 2012; Bricker *et al*., 2018; Edgeloe *et al*., 2022), but still striking if taking into account the small plant size of a *N. japonica* ramet.

In conclusion, we show the existence of two cryptic species within the seagrass *N. japonica*, using whole‐genome sequencing data and a dated molecular clock approach. A formal taxonomic description would be our next step and will entail sampling voucher specimen, including their floral organs, carry out histological sections and depositing those in museum collections, along with additional morphological and histological descriptions and genotyping. This study indicates that the true diversity of marine angiosperms or seagrasses is likely to be underestimated due to cryptic species‐level diversity. Given the long‐term bias of cryptic diversity studies on higher plants (Bickford *et al*., 2007; Struck *et al*., 2018), additional efforts are needed for re‐evaluating the species diversity of seagrasses as well as other vascular plants, aided by the increasing availability of population‐level genome data. Using such approaches, we expect that there may be more seagrasses species discovered in the future, in particular in species with a broad latitudinal distribution. An interesting question is why some extremely widespread species, such as the emerging model seagrass species *Z. marina* seems to be one cohesive species, despite its distributional range occupying the temperate areas along both coastlines of Northern hemisphere Pacific and Atlantic oceans (Yu *et al*., 2023). Whether this may be attributable to the long‐distance dispersal potential of *Z. marina* caused by the abiotic (Harwell & Orth, 2002) or biotic (Sumoski & Orth, 2012) drivers is unresolved. Notwithstanding, identification of cryptic macrophyte species is important for a more comprehensive recognition and conservation of coastal biodiversity and science‐guided strategies on their protection and management.

## Competing interests

None declared.

## Author contributions

XZ, Y Zhou, and TBHR conceived and designed this study; XZ, ZS, TK, GQ, SX, MX, FW, SY, K‐SL, and Y Zhang conducted sampling; Y‐LL and J‐XL assembled the genomes; XZ conducted SSR genotyping and karyotyping analyses; LY conducted the SNP calling and all the other analyses; XZ, LY, and TBHR discussed and interpreted the results; XZ, LY, Y‐LL, and TBHR wrote the paper. All authors commented on earlier versions of the manuscript. XZ, LY, and Y‐LL contributed equally to this work.

## Disclaimer

The New Phytologist Foundation remains neutral with regard to jurisdictional claims in maps and in any institutional affiliations.

## Supporting information


**Dataset S1** Genotypes for the triploid genets based on 17 microsatellite loci.
**Dataset S2** Ploidy determination using nine microsatellite loci.

**Fig. S1** Chromosome‐level reference genome for the seagrass *Nanozostera japonica* (northern clade, Nj_N).
**Fig. S2** Busco scores for six seagrass genomes.
**Fig. S3** The geographic distribution of the 19 clones with ≥ 2 ramets listed in Table S4.
**Fig. S4** Triangle plot for hybrid index and interclass heterozygosity.
**Fig. S5** Admixture in the *Nanozostera japonica* at the contact zone.
**Fig. S6** Chromosome‐level reference genome for southern clade of the seagrass *Nanozostera japonica* (Nj_S).
**Fig. S7** PCA plot for the first PC (PC1) based on the 131 306 SNPs located in the inversion region (Nj_N, Chr04: 55648726–96617151) for all the unique genets.
**Fig. S8** Morphological representatives for the two genetic clades (Nj_N and Nj_S) of *Nanozostera japonica*.
**Fig. S9** Comparison of morphological measurements for Nj_N and Nj_S.
**Methods S1** Detailed materials and methods.
**Table S1** Information for the chromosome‐level reference genomes of *Nanozostera japonica* assembled in this study.
**Table S2** Gene prediction and annotation for the chromosome‐level reference genome of *Nanozostera japonica* assembled in this study.
**Table S3** Sampling information of *Nanozostera japonica* across its range in Northwestern Pacific.
**Table S4** Information for the *Nanozostera japonica* genets with > 2 ramets and their original populations.
**Table S5** Comparison of morphological measurements for Nj_N and Nj_S.
Please note: Wiley is not responsible for the content or functionality of any Supporting Information supplied by the authors. Any queries (other than missing material) should be directed to the *New Phytologist* Central Office.

## Data Availability

The assembled Nj_N reference genome, including the relevant sequencing data, has been deposited in NCBI under the accession no. PRJNA1130640, and the annotation files are available in figshare (doi: 10.6084/m9.figshare.26500192). The assembled Nj_S reference genome, including the relevant sequencing data, has been deposited in NCBI under the accession no. PRJNA1153406, and the annotation files are available in figshare (doi: 10.6084/m9.figshare.26878903). Custom‐made scripts are deposited on Github (https://github.com/leiyu37/PopulationGenomics_Nj). Whole‐genome resequencing data have been deposited in Genbank short read archive: 2 *Z. marina* seedlings, SRR26434424 and SRR26434425; 17 *N. noltii* ramets, SRR29681770–SRR29681786; 307 *N. japonica* ramets, SRR29665291–SRR29665315, SRR29687066–SRR29687104, SRR29687107–SRR29687125, SRR29702327–SRR29702350, SRR29702359–SRR29702398, SRR29708351–SRR29708409, SRR29721242–SRR29721279, SRR29721358–SRR29721375, SRR29726569–SRR29726588, SRR29726676–SRR29726700.
