# Supplementary material for: Uncovering the Nanozostera japonica species complex suggests cryptic speciation and underestimated seagrass diversity
Source: New Phytol. 2025 Jun 30;247(5):2086–97. doi: 10.1111/nph.70355 (PMC12329173; doi:10.1111/nph.70355)
Supplement: Supplementary file 2 — Fig. S1 Chromosome‐level reference genome for the seagrass Nanozostera japonica (northern clade, Nj_N). Fig. S2 Busco scores for six seagrass genomes. Fig. S3 The geographic distribution of the 19 clones with ≥ 2 ramets listed in Table S4. Fig. S4 Triangle plot for hybrid index and interclass heterozygosity. Fig. S5 Admixture in the Nanozostera japonica at the contact zone. Fig. S6 Chromosome‐level reference genome for southern clade of the seagrass Nanozostera japonica (Nj_S). Fig. S7 PCA plot for the first PC (PC1) based on the 131 306 SNPs located in the inversion region (Nj_N, Chr04: 55648726–96617151) for all the unique genets. Fig. S8 Morphological representatives for the two genetic clades (Nj_N and Nj_S) of Nanozostera japonica. Fig. S9 Comparison of morphological measurements for Nj_N and Nj_S. Methods S1 Detailed materials and methods. Table S1 Information for the chromosome‐level reference genomes of Nanozostera japonica assembled in this study. Table S2 Gene prediction and annotation for the chromosome‐level reference genome of Nanozostera japonica assembled in this study. Table S3 Sampling information of Nanozostera japonica across its range in Northwestern Pacific. Table S4 Information for the Nanozostera japonica genets with > 2 ramets and their original populations. Table S5 Comparison of morphological measurements for Nj_N and Nj_S. Please note: Wiley is not responsible for the content or functionality of any Supporting Information supplied by the authors. Any queries (other than missing material) should be directed to the New Phytologist Central Office. [file NPH-247-2086-s001.pdf]

New Phytologist Supporting Information

Article title: Uncovering the *Nanozostera japonica* species complex suggests cryptic speciation and underestimated seagrass diversity

Authors: Xiaomei Zhang, Lei Yu, Yu-Long Li* Zhaxi Suonan, Teruhisa Komatsu, Guanglong Qiu, Shaochun Xu, Shidong Yue, Min Xu, Feng Wang, Yu Zhang, Kun-Seop Lee, Jin-Xian Liu, Yi Zhou, Thorsten B.H. Reusch

Article acceptance date: 09 June 2025

The following Supporting Information is available for this article:

Fig. S1 Chromosome-level reference genome for the seagrass *Nanozostera japonica* (northern clade, Nj_N).

Fig. S2 BUSCO scores for six seagrass genomes.

Fig. S3 The geographic distribution of the 19 clones with >= 2 ramets listed in Table S4.

Fig. S4 Triangle plot for hybrid index and interclass heterozygosity.

Fig. S5 Admixture in the *Nanozostera japonica* at the contact zone.

Fig. S6 Chromosome-level reference genome for southern clade of the seagrass *Nanozostera japonica* (Nj_S).

Fig. S7 PCA plot for the first PC (PC1) based on the 131,306 SNPs located in the inversion region (Nj_N, Chr04: 55648726-96617151) for all the unique genets.

Fig. S8 Morphological representatives for the two genetic clades (Nj_N (a-c) and Nj_S (d-f)) of *Nanozostera japonica*.

Fig. S9 Comparison of morphological measurements for Nj_N and Nj_S.

Table S1 Information for the chromosome-level reference genomes of *Nanozostera japonica* assembled in this study.

Table S2 Gene prediction and annotation for the chromosome-level reference genome of *Nanozostera japonica* assembled in this study.

Table S3 Sampling information of *Nanozostera japonica* across its range in Northwestern Pacific.

Table S4 Information for the *Nanozostera japonica* genets with more than 2 ramets and their original populations.

Table S5 Comparison of morphological measurements for Nj_N and Nj_S.

Methods S1

Fig. S1 Chromosome-level reference genome for the seagrass *Nanozostera japonica* (northern clade, Nj_N). a, Circos plot for the reference genome. Tracks from inside to outside: genome synteny across chromosomes, density of SNPs, density of genes, density of transposable elements (TE) and GC content. b, Plot of HiC heatmap. The colour indicates the strength of the chromosomal interaction between different regions of the genome.

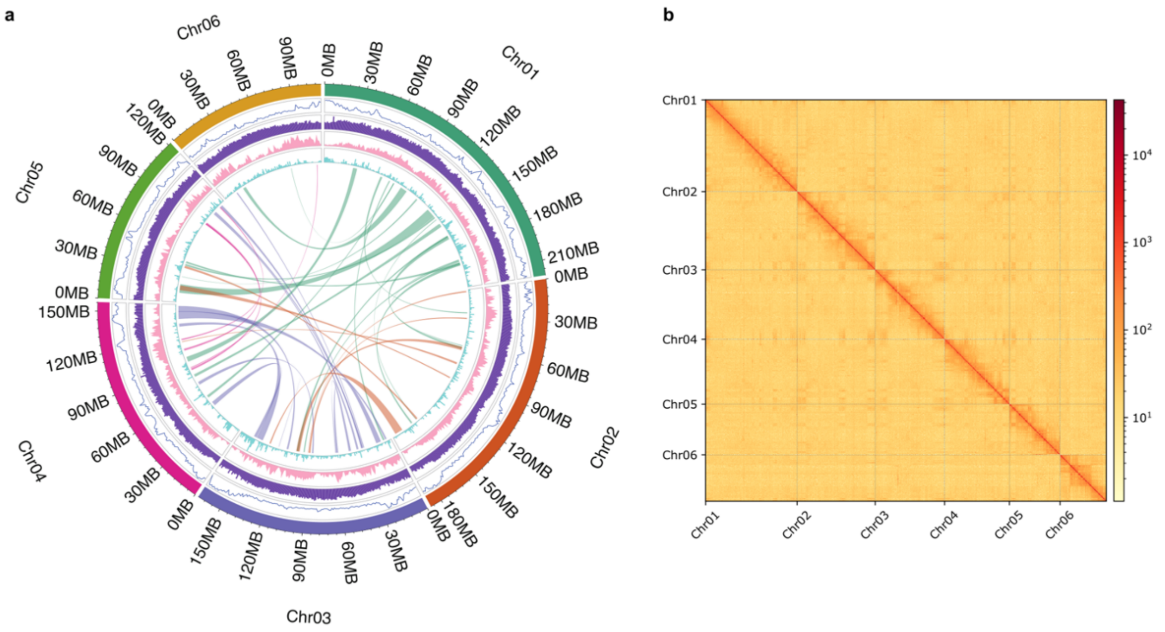

Fig. S2 BUSCO scores for six seagrass genomes. *Cymodocea nodosa*[31], *Nanozostera japonica* (assembled in this study), *Posidonia oceanica*[31], *Thalassia testudinum*[31], and *Zostera marina*[24] based on embryophyta_odb10 database.

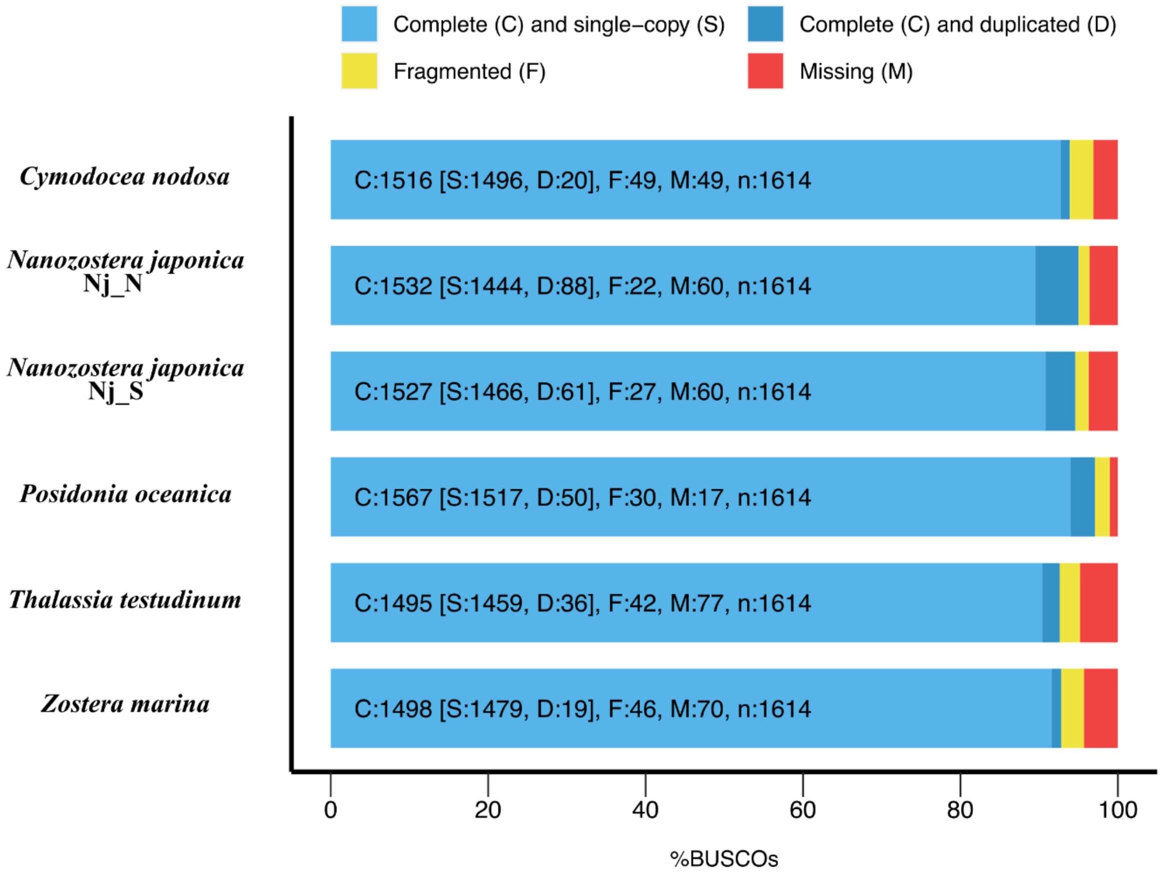

Fig. S3 The geographic distribution of the 19 clones with >= 2 ramets.

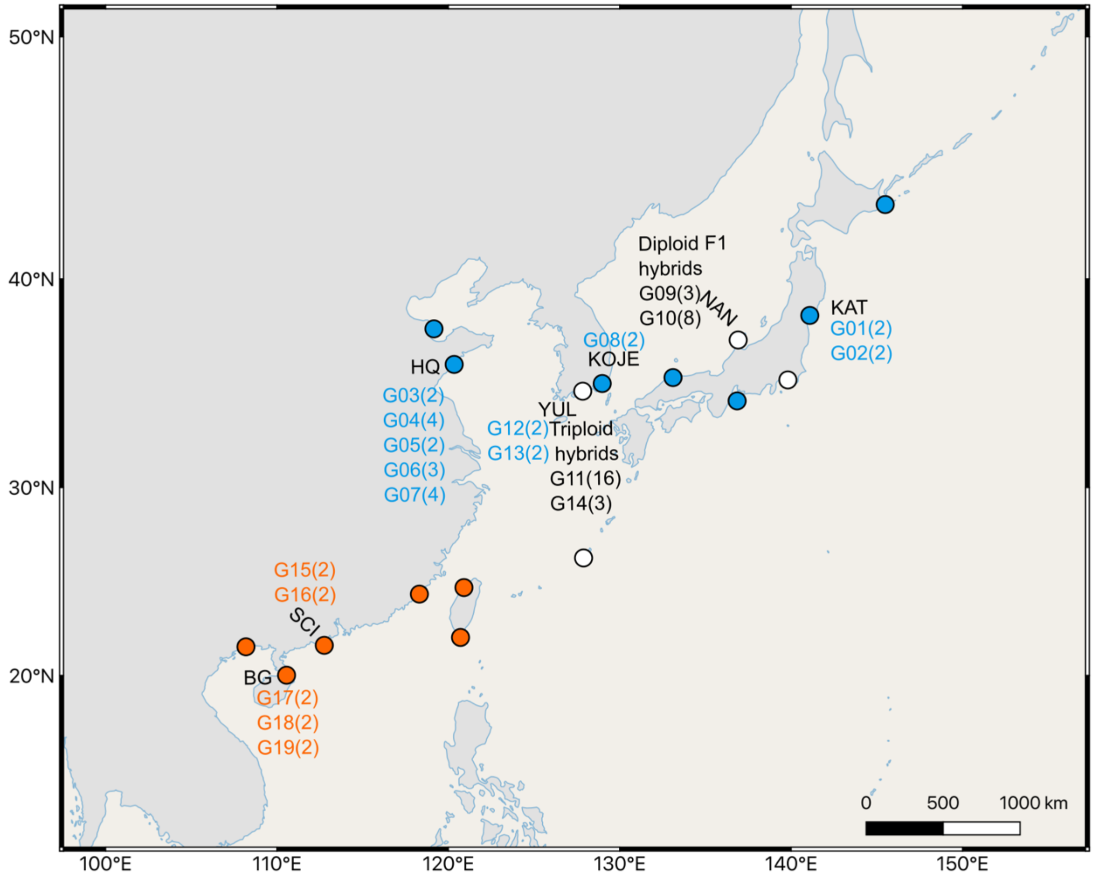

Fig. S4 Triangle plot for hybrid index and interclass heterozygosity. The plot is based on 58,029 SNPs with fixed differences between Nj_N and Nj_S (pairwise distance between SNPs > 3000 bp). The green and purple dots contain 135 and 115 samples, respectively. Since all samples within the same clade overlap with each other, only one dot is visible.

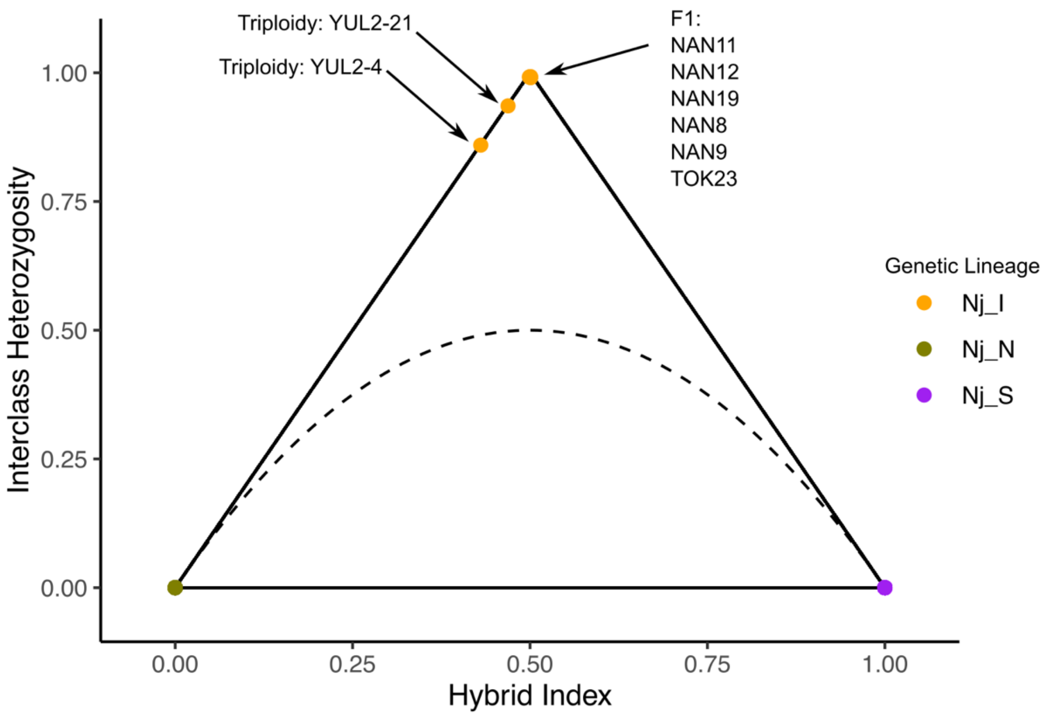

Fig. S5 Admixture in the *Nanozostera japonica* at the contact zone. The eight genets in the intermediate group of PCA, NJ tree, and STRUCTURE analyses are shown. The ratio of the two genetic components is based on the STRUCTURE analyses, which is almost equal to 1:1 for the first six genets.

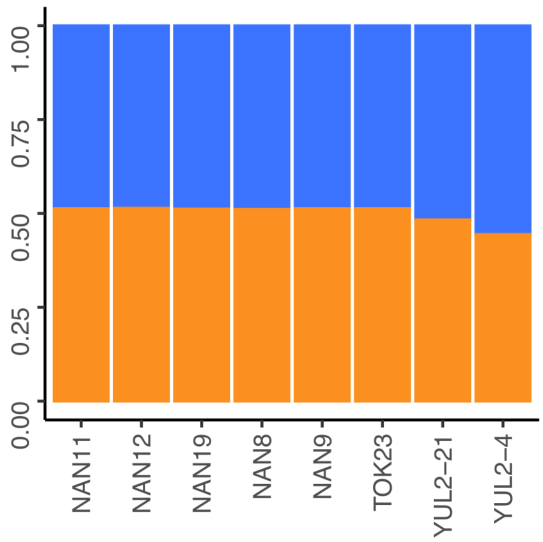

Fig. S6 Chromosome-level reference genome for southern clade of the seagrass *Nanozostera japonica* (Nj_S). a, Circos plot for the reference genome. Tracks from inside to outside: genome synteny across chromosomes, density of SNPs, density of genes, density of transposable elements (TE) and GC content. b, Plot of HiC heatmap. The colour indicates the strength of the chromosomal interaction between different regions of the genome.

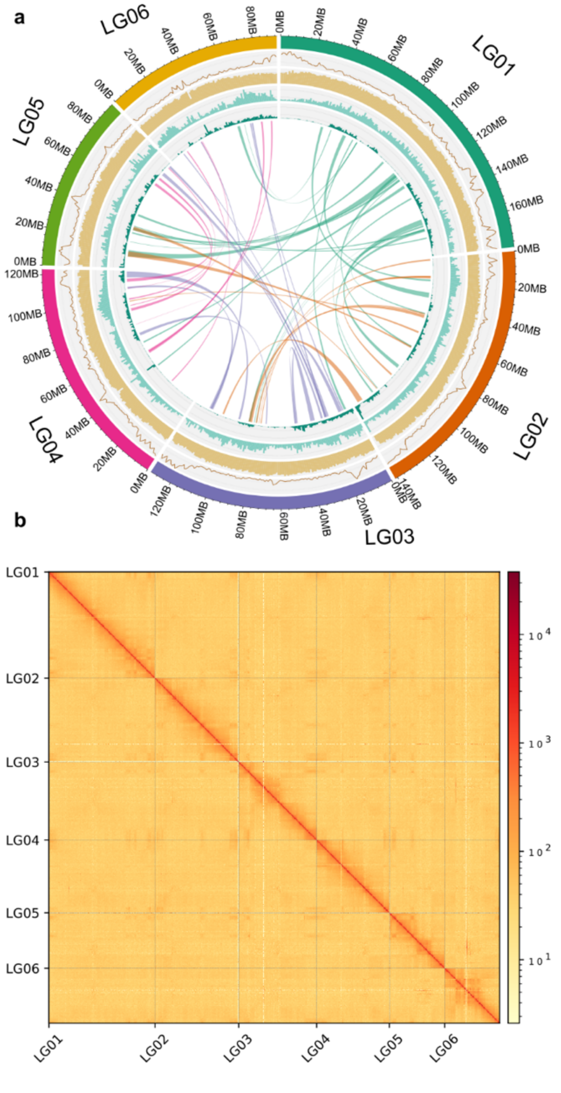

Fig. S7 PCA plot for the first PC (PC1) based on the 131,306 SNPs located in the inversion region (Nj_N, Chr04: 55648726-96617151) for all the unique genets. The leftmost and the rightmost groups represent the homozygous states for the inversion, while the middle group indicates heterozygosity. Individuals of Nj_N are distributed exclusively in the leftmost group, while individuals of Nj_S are distributed exclusively in the rightmost group. This indicates that Nj_N and Nj_S are fixed with different inversion states. In addition, both F1 hybrids and triploid hybrids show heterozygosity for the chromosomal inversion.

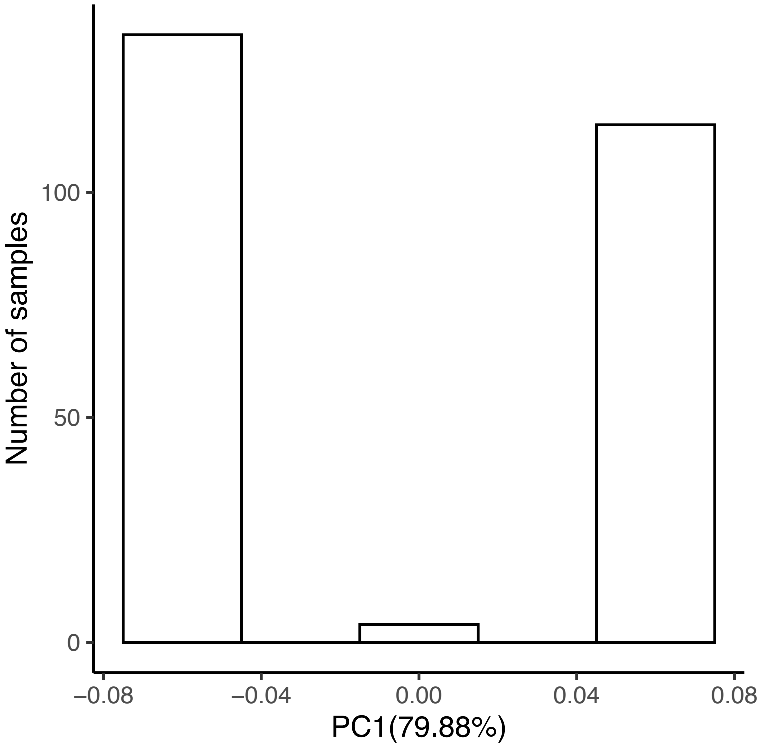

Fig. S8 Morphological representatives for the two genetic clades (Nj_N (a-c) and Nj_S (d-f)) of *Nanozostera japonica*. Almost all the morphological characteristics, except the ones lacking enough data (e.g., spathe length), show higher variation within clades than between clades.

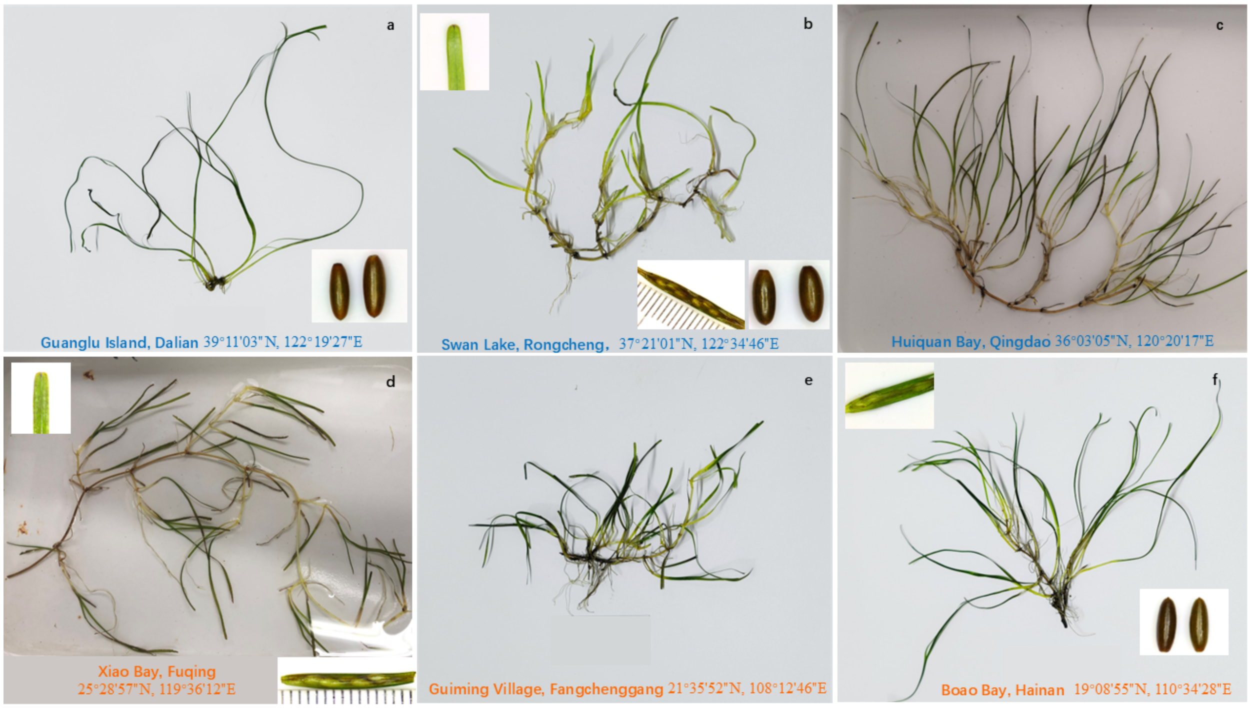

Fig. S9 Comparison of morphological measurements for Nj_N and Nj_S.

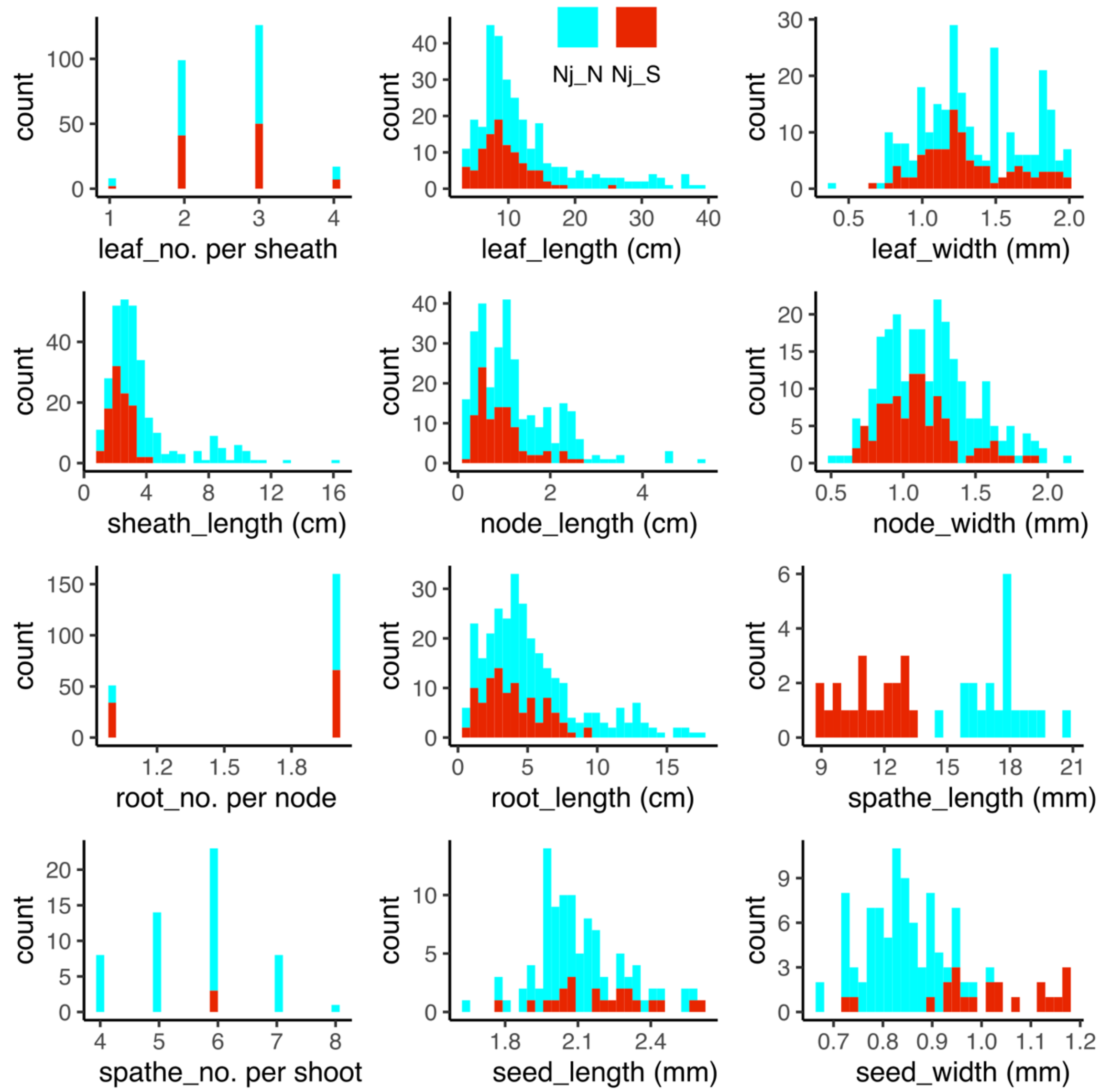

Table S1 Information for the chromosome-level reference genomes of *Nanozostera japonica* assembled in this study.

BUSCO scores for seagrass genomes					
Species	Complete BUSCOs	Complete& single-copy BUSCOs	Complete & duplicated BUSCOs	Fragmented BUSCOs	Missing BUSCOs
Nanozostera japonica **Nj-N**	1532	1444	88	22	60
Nanozostera japonica **Nj-S**	1527	1466	61	27	60
Cymodocea nodosa	1516	1496	20	49	49
Posidonia oceanica	1567	1517	50	30	17
Thalassia testudinium	1495	1459	36	42	77
Zosma marina	1498	1479	19	46	70

Assembly metrics for seagrass genomes								
Species	n_scaffolds	n_contigs	scaf_bp	contig_bp	scaf_N50	ctg_N50	scaf_N90	ctg_N90
Nanozostera japonica **Nj-N**	887	920	1,052,869,351	1,052,866,046	167,191,925	52,285,153	111,875,394	3,452,612
Nanozostera japonica **Nj-S**	378	412	805,155,086	805,151,687	131,576,194	48,988,836	92,951,072	11,134,760
Cymodocea nodosa	23	23	379,522,331	379,522,331	20,493,075	20,493,075	11,945,794	11,945,794
Posidonia oceanica	81	81	2,962,985,811	2,962,985,811	67,525,845	67,525,845	48,835,270	48,835,270
Thalassia testudinium	240	240	4,261,934,351	4,261,934,351	111,514,854	111,514,854	111,514,854	111,514,854
Zosma marina	2228	12588	203,913,826	191,658,944	485,578	79,958	95,502	9,156

Table S2 Gene prediction and annotation for the chromosome-level reference genome of *Nanozostera japonica* assembled in this study. Genes were predicted using the evidence from both ab initio gene predictors (Augustus, GeneMark, Snap), protein (MetaEuk) and transcript alignments (Minimap2+StringTie, PASApipeline), and were finally refined using EVidenceModeler, Funannotate and PASApipeline. The predicted genes were annotated using public data bases, including Swiss-Prot/TrEMBL, Pfam, EggNOG, and InterProScan.

Gene prediction	Nj-N			Nj-S		
	No. of genes	No. of exons	No. of introns	No. of genes	No. of exons	No. of introns
Augustus	41203	129511	88308	24629	85519	60890
GeneMark	35791	144048	108257	44164	166960	122796
Snap	250828	552640	301812	199768	449294	249526
Braker	37136	261806	222366	40035	168909	127133
Minimap2+StringTie	-	369189	-	-	182384	-
MetaEuk	-	171127	-	-	108659	-
PASApipeline	16083	91685	75602	15044	81923	66879
EVidenceModeler+Funannotate	21574	126552	104978	21912	120591	98679
PASApipeline update UTR	22074	128046	105972	22284	122311	100027

Gene annotation database	No. of genes	
	Nj-N	Nj-S
Pfam	15774	14966
InterPro	18523	18094
EggNOG	19925	19723
COG	18985	18719
GO Terms	13486	15321
Swiss-Prot/TrEMBL	20319	19994
Total annotated	20421	20394

Table S3 Sampling information of *Nanozostera japonica* across its range in Northwestern Pacific. N_{sample}, the number of the samples collected for each population; N_{ramet}, the number of ramets successfully sequenced for each population; N_{genet}, the number of genets for each population.

	**Code**	**Country**	**Location**	**Latitude**	**Geogr. distance between sample collection**	N_{sample}	N_{ramet}	N_{genet}
**1**	HQ	China	Huiquan Bay, Qingdao	36°03'05"N, 120°20'17"E	>2m	25	25	15
**2**	DY	China	Yellow River Delta, Dongying	37°43'09"N, 119°09'49"E	~20m	20	20	20
**3**	KOJE	South Korea	Yongbuk, Koje Island, Busan	35°26'58"N, 128°35'45"E	>10m	6	6	5
**4**	OHA	Japan	Ohashi River, Shimane	34°48'04"N, 133°06'45"E	⩾10m	16	16	16
**5**	ONN	Japan	Onneto Lake, Nemuro, Hokaido	43°15'47"N, 145°29'28"E	~10m	20	20	20
**6**	KAT	Japan	Katsura-jima Island, Miyagi	38°20'08"N, 141°05'14"E	>5m	17	17	15
**7**	TOK	Japan	Tokyo Bay, Tokyo	35°18'46"N, 139°48'49"E	~10m	20	20	20
**8**	AGO	Japan	Ago Bay, Mie	34°18'03"N, 136°50'50"E	~10m	20	20	20
**9**	NAN	Japan	Nanao bay, Noto Penisula	37°12'11"N, 136°54'24"E	2-10m	18	16	7
**10**	YUL	South Korea	Yulpo, Koje Island, Busan	34°46'04"N, 128°35'45"E	5-10 m	25	25	6
**11**	OKI	Japan	Okinawa Island	26°20'51"N, 127°53'07"E	2-5m	18	17	17
**12**	WJR	China	Wujiang River, Kinmen Island	24°25'36"N, 118°18'34"E	≥5m	20	20	20
**13**	XSW	China	Xiangshan Wetland, Taiwan Island	24°46'38"N, 120°54'45"E	>5m	20	20	20
**14**	GXPD	China	Pingdong, Gaoxiong, Taiwan Island	22°05'17"N, 120°42'35"E	~10m	9	9	9
**15**	SCI	China	Shangchuandao Island, Taishan	21°39'22"N, 112°45'48"E	~1m	15	15	13
**16**	GXGM	China	Guiming village, Fangchenggang	21°35'52"N, 108°12'46"E	5-20m	19	19	19
**17**	BG	China	Beigang Island, Haikou	20°00'54"N, 110°33'45"E	10-20m	19	19	16
	Total					307	304	258

Table S4 Information for the *Nanozostera japonica* genets with more than 2 ramets and their original populations.

Population	Location	N_{ramet}	N_{genet}	R	Genet ID	Clone mates	Identity
KAT	Katsura-jima Island, Miyagi, Japan	17	15	0.88	1	Kat10<br>Kat11	Diploid Nj-N
					2	Kat5<br>Kat8	Diploid Nj-N
HQ	Huiquan Bay, Qingdao, China	25	15	0.60	3	HQ10-10<br>HQ10-11	Diploid Nj-N
					4	HQ10-20<br>HQ10-21<br>HQ10-23<br>HQ10-24	Diploid Nj-N
					5	HQ10-5<br>HQ10-6	Diploid Nj-N
					6	HQ4-4<br>HQ6-11<br>HQ6-9	Diploid Nj-N
					7	HQ6-1<br>HQ6-3<br>HQ6-5<br>HQ6-6	Diploid Nj-N
KOJE	Yongbuk, Koje Island, Busan, South Korea	6	5	0.83	8	Koje-1-5<br>Koje02	Diploid Nj-N
NAN	Nanao bay, Noto Penisula, Japan	16	7	0.44	9	Nan13<br>Nan14<br>Nan9	Diploid hybrid F1
					10	Nan16<br>Nan19<br>Nan23<br>Nan29<br>Nan30<br>Nan32<br>Nan34<br>Nan35	Diploid hybrid F1
YUL	Yulpo, Koje Island, Busan, South Korea	25	6	0.24	11	Yul-1-17<br>Yul-2-15<br>Yul-2-18<br>Yul1-13<br>Yul1-14<br>Yul1-15<br>Yul1-16	Triploid hybrid

						Yul2-10<br>Yul2-14<br>Yul2-19<br>Yul2-20<br>Yul2-21<br>Yul2-22<br>Yul2-5<br>Yul2-8<br>Yul2-9	
					12	Yul-1-19<br>Yul2-12	Diploid Nj-N
					13	Yul-2-16<br>Yul2-17	Diploid Nj-N
					14	Yul2-3<br>Yul2-4<br>Yul2-6	Triploid hybrid
SCI	Shangchuandao Island, Taishan, China	15	13	0.87	15	SCI21<br>SCI22	Diploid Nj-S
					16	SCI24<br>SCI28	Diploid Nj-S
BG	Beigang Island, Haikou, China	19	16	0.84	17	BG14<br>BG21	Diploid Nj-S
					18	BG20<br>BG6	Diploid Nj-S
					19	BG24<br>BG3	Diploid Nj-S

Table S5 Comparison of morphological measurements (±SD) for Nj_N and Nj_S. Samples for Nj-N were collected in August 2015 or September 2024, and the samples for Nj-S were collected in October 2024.

Group	Location		Leaf			Sheath	Node	
			No. per sheath	length(cm)	width(mm)	length(cm)	length(cm)	width(mm)
Nj-N	Guanglu Island, Dalian	39°11'03"N, 122°19'27"E	2.50±0.54	24.64±8.53	1.57±0.30	2.50±0.54	0.52±0.47	1.32±0.36
	Yellow River Delta, Dongying	37°43'09"N, 119°09'49"E	3.16±0.42	13.27±4.06	1.55±0.32	3.16±0.42	3.40±0.79	1.79±0.85
	Swan Lake, Rongcheng	37°21'01"N, 122°34'46"E	2.14±0.61	8.44±3.19	1.02±0.22	2.14±0.61	1.66±0.65	0.96±0.21
	Huiquan Bay, Qingdao	36°03'05"N, 120°20'17"E	-	8.28±2.22	1.26±0.20	2.93±0.87	1.40±1.09	1.39±0.23
Nj-S	Xiao Bay, Fuqing	25°28'57"N, 119°36'12"E	-	-	-	-	-	-
	Guiming Village, Fangchenggang	21°35'52"N, 108°12'46"E	2.60±0.70	7.27±2.04	1.41±0.29	2.21±0.21	1.06±0.62	1.11±0.29
	Boao Bay, Hainan	19°08'55"N, 110°34'28"E	2.64±0.60	11.31±3.58	1.21±0.31	2.64±0.60	0.76±0.33	1.11±0.24

Group	Location		Root		Spathe		Seed	
			No. per node	length(cm)	length(mm)	No. per shoot	length(mm)	width(mm)
Nj-N	Guanglu Island, Dalian	39°11'03"N, 122°19'27"E	1.76±0.43	2.86±1.90	-	5.09±0.94	2.27±0.14	0.84±0.05
	Yellow River Delta, Dongying	37°43'09"N, 119°09'49"E	-	-	-	-	2.02±0.09	0.86±0.08
	Swan Lake, Rongcheng	37°21'01"N, 122°34'46"E	1.90±0.30	3.98±1.07	17.50±1.41	5.95±0.76	2.06±0.16	0.79±0.07
	Huiquan Bay, Qingdao	36°03'05"N, 120°20'17"E	2.00±0.00	6.52±2.61	-	5.55±1.15	2.00±0.15	0.84±0.06
Nj-S	Xiao Bay, Fuqing	25°28'57"N, 119°36'12"E	-	-	11.20±1.42	6.00±0.00	2.21±0.21	1.04±0.10
	Guiming Village, Fangchenggang	21°35'52"N, 108°12'46"E	1.76±0.43	4.62±2.11	-	-	-	-
	Boao Bay, Hainan	19°08'55"N, 110°34'28"E	1.56±0.50	3.18±1.73	-	-	2.03±0.01	0.74±0.01

Methods S1

Seagrass *Nanozostera japonica*

Nanozostera japonica is one of the few seagrass species distributed across both tropical and temperate zones in the world, displaying highly plastic morphological traits and life history strategies among different geographical locations. Thus, it provides a good model to examine the potential cryptic speciation under such wide range of environment gradients. *N. japonica* has been widely called *Zostera japonica* in the ecological literature Here, we adopted the genus status of *Nanozostera* proposed repeatedly in recent taxonomic work[1 2]. Especially, convincing evidence for four genera within the family Zosteraceae was provided based on molecular markers and molecular genetic distances among the 4 genera[3].

Genome assembly

We assembled two chromosome-level reference genomes for the northern and southern distributional range, representing Nj_N and Nj_S, respectively. For the Nj_N reference genome, samples were collected from Huiquan Bay, Qingdao, northern China (36°03’05”N, 120°20’17”E). As for the Nj_S reference genome, samples were collected from Pearl Bay, Fangchenggang, southern China (21°35’52”N, 108°12’46”E). The samples were carefully cleaned and subsequently sent to Biomarker Technologies (Qingdao, China) for DNA and RNA extraction. High-quality genomic DNA was extracted from whole shoots of the same genet using a modified CTAB method. DNA concentration and quality were determined using a NanoDrop 2000 spectrophotometer (Thermo Fisher Scientific, Waltham, MA, USA), a Qubit fluorimeter (Invitrogen, QubitTM3Flurometer), and 1.0% agarose gel electrophoresis. Total RNA was extracted from multiple tissues including leaves and sheaths, rhizomes, and roots using a RNAprep plant kit (TIANGEN, Beijing). Following QC was conducted using a NanoDrop 2000 spectrophotometer, an Agient 2100 bioanalyzer instrument, and a LabChip GX touch nucleic acid analyzer. Genomic DNA libraries were prepared according to the manufacturer’s instructions. In brief, genomic DNA was sheared into ~ 15 kb fragments by Megaruptor 2. The SMRTbell library was constructed using the SMRTbell Express Template Prep kit 2.0 (Pacific Biosciences), which was then sequenced using the PacBio Sequel II System. In addition, short-insert (350 bp) paired-end library was constructed and sequenced on Illumina NovaSeq 6000 platform. Hi-C library was constructed following the standard protocols (VAHTSTM Fg DNA Library Prep Kit, Vazyme, China) and sequenced on Illumina NovaSeq 6000 platform.

PacBio long sequence reads were first converted into adaptor-removed subreads. The subreads were error corrected by CANU v2.2[4], and they were then assembled into contigs by SMARTDENOVO v1.0[5]. The contigs were polished three rounds by PILON v1.23[6] based on high-quality Illumina reads. These contigs were anchored into pseudo-chromosomes based on HiC data by LACHESIS[7] using these parameters:
CLUSTER_MIN_RE_SITES=89;
CLUSTER_MAX_LINK_DENSITY=2;
LUSTER_NONINFORMATIVE_RATIO=2;
ORDER_MIN_N_RES_IN_TRUN=56;
ORDER_MIN_N_RES_IN_SHREDS=56.

Scaffolds were gap-filled using TGS-GAPCLOSER v1.2.0[8] with PacBio long sequence reads and Illumina reads to improve the continuity, with a minimum of three reads to bridge the gap. The gap-filled scaffolds were finally corrected using GCPP v2.0.2 (https://github.com/PacificBiosciences/gcpp) with PacBio long reads, and they were then polished using FREEBAYES v1.3.4[9] and PILON v1.2.3 with high-quality Illumina reads (PE150, Illumina NovaSeq 6000).

To assess the assembly quality, the Illumina reads were aligned to the assembled chromosome-level reference genome using BWA v0.7.17[10], and the alignment metrics including mapping rate, proper mapping rate were calculated by SAMTOOLS v1.10[11]. BUSCO v5.3.2[12] was used to evaluate genome completion based on the 1,614 embryophyta gene dataset with default parameters.

Gene prediction and annotation

Genes were predicted using the evidence from both ab initio gene predictors and protein and transcript alignments. Firstly, a de novo repeat library was constructed using EDTA v2.0.0[13] and REPEATMODELER v2.0.1[14], and repeats were identified using REPEATMASKER v4.1.2 (https://www.repeatmasker.org/). Repeat sequences excluding low-complexity components were soft masked. Secondly, BRAKER v2.1.6[15] was used to train the gene prediction tools GENEMARK v4.69[16] and AUGUSTUS v3.4.0[17], and the ab initio predictions were generated based on RNA-seq data and protein homology information of plants from OrthoDB database. Thirdly, RNA-seq reads were assembled using TRINITY v2.11.0[18], and the results were passed to PASAPIPELINE v2.4.1[19] to generate high quality gene structures. Fourthly, PacBio long read RNA sequencing reads were clustered into transcripts by PacBio IsoSeq3 pipeline, together with transcripts assembled by TRINITY, were used to generate evidence from RNA alignments by MINIMAP2 v2.17-r941[20] and STRINGTIE v2.2.1[21]. Fifthly, evidence from protein sequences of *Zostera marina* from Phytozome database were extracted from the alignments to the genome by using METAEUK v57b63975a942fbea328d8ea39f620d6886958eca[22]. Finally, the above ab initio gene predictions, protein and transcript alignments were combined into weighted consensus gene structures using EVIDENCEMODELER v1.1.1[19] accomplished by FUNANNOTATE v1.8.10 (https://github.com/nextgenusfs/funannotate). Protein functions were annotated using Funannotate v1.8.15 from alignments on multiple databases including the Swiss-Prot/TrEMBL, Pfam, EggNOG, and InterProScan.

Large-scale sample collection

Samples were collected from the native range of *N. japonica*. We considered the multiple shoots connected by the same rhizome as one sample, and they were collected by walking or wading during the low tide. The distance between samples varied from 30 cm to >20 m among different locations, depending on the seagrass meadow status, e.g., area, continuous vs. patchy, etc. Fresh leaves, sheaths, and rhizomes were preserved in silica gel or in plastic bags in a cooler on ice in the field and then stored at -80°C until DNA extraction. High-quality genomic DNA was extracted using CTAB method.

Seventeen *N. noltii* samples were collected from Germany (54°40’57”N, 9°59’47”E) by snorkeling. The samples were kept in seawater for a few hours until being processed in the lab. Then, the

samples were stored at -80°C until DNA extraction. High-quality genomic DNA was extracted from whole shoots using the Macherey-Nagel NucleoSpin plant II kit following the manufacturer's instructions.

Two seedlings of *Zostera marina* collected from the field were cultured in the lab until they were large enough for DNA extraction. Entire plants except roots were cleaned and processed, which were immediately used for DNA extraction (Macherey-Nagel NucleoSpin plant II kit).

Whole-genome resequencing

We conducted whole-genome resequencing for 307 *N. japonica* samples from 17 populations (Fig.1a and Supplementary table1) throughout its biogeographic range in the North-Western Pacific, 17 *N. noltii* samples from Germany, and 2 *Z. marina* seedlings cultured in the lab. Paired-end libraries with an insert size of ~350 bp were constructed according to the Illumina or BGI instructions, which were then sequenced on the Illumina Novaseq6000 or BGI DNBSEQ-T7, targeted at a depth of ~20× coverage for *N. japonica* and *N. noltii* while 1000× for *Z. marina*.

SNP calling and filtering

The quality of the raw reads was assessed using FastQC v0.11.7 (https://www.bioinformatics.babraham.ac.uk/projects/fastqc/). Raw reads from different sequencing rounds were merged by Linux command "zcat". BBDuk (https://jgi.doe.gov/data-and-tools/software-tools/bbtools/bb-tools-user-guide/bbduk-guide/) was used to remove adapters and for quality filtering according to the following criteria: (1) sequence downstream with quality <20 was trimmed (trimq = 20); (2) reads shorter than 50 bp after trimming were discarded (minlen = 50); (3) reads with average quality below 20 after trimming were discarded (maq = 20). FastQC was used to do a second round of quality check for the clean reads. Clean reads were then mapped against the assembled Nj_N reference genome using BWA-MEM v0.7.17 with default parameters. The aligned reads were sorted using SAMtools v1.7, and duplicated reads were marked using MarkDuplicates tool in GATK v4.0.1.2. Only properly paired reads (0 × 2) with MAPQ of at least 20 (-q 20) were kept using SAMtools.

GATK4 was used to conduct joint SNP calling. In brief, HaplotypeCaller was used to generate a GVCF-format file for each bam-format file, and GenotypeGVCFs was used for SNP calling based on the combined GVCF file generated by CombineGVCFs. VariantsToTable (GATK4) was used to extract INFO annotations (CHROM, POS, FILTER, QD, MQ, FS, SOR, MQRankSum, ReadPosRankSum, and DP). SNPs were marked by VariantFiltration (GATK4), which were further cleaned by SelectVariants (GATK4). VCFtools was used to convert individual genotypes to missing data when GQ < 30 or DP < 10 and keep only bi-allelic SNPs. Genotypes that were outside our custom quality criteria were represented as missing data. For each sample, the missing rate was calculated using a custom python script, i.e., number of heterozygous loci / total number of loci with available genotypes. Three samples (NAN6, NAN7, and OKI24) had missing genotypes at >90% of the loci. VCFtools was then used to keep only polymorphic SNPs based on the remaining available genotypes, and then NAN6, NAN7, and OKI24 were removed. VCFtools v0.1.13 was used again to keep only polymorphic SNPs based on the remaining available genotypes, and only SNPs with missing rate <0.1 were kept.

Joint SNP calling and filtering were conducted separately for *N. noltii* using the similar method mentioned above. The following thresholds were used for hard filtering: QD < 10.0, FS > 60.0, SOR > 3.0, MQ < 40.0, MQRankSum > 2.5, MQRankSum < -2.5, ReadPosRankSum > 2.5, ReadPosRankSum < -2.5, DP > 873.88.

Detecting clone mates (ramets) and genets

After filtering, the shared heterozygosity method[23] was used to identify clonemate pairs. However, we found that hybrids shared extremely high levels of heterozygosity (>0.9), due to the large number of fixed differences between genetic clades. Since clonemate pairs were supposed to rarely show fixed differences, i.e., different homozygous genotypes (N_homoDiff), due to the absence of recombination in clonal reproduction, we decided to use the pairwise fixed differences as an additional index for clonemate detection. Clone detection was done separately for *N. japonica* and *N. noltii*.

For each genet, only the ramet with the lowest data missing rate was kept, while all redundant ramets were removed. Accordingly, a total of 46 samples were removed for *N. japonica*. VCFtools was used to keep only polymorphic SNPs based on the remaining available genotypes, and only SNPs with missing rate <0.1 were kept. Similarly, the 17 *N. noltii* ramets were found to belong to 3 unique genets, and redundant ramets within the same genet were also excluded.

Genetic population structure

Principal component analysis (PCA) and Neighbor-joining tree were based on the Core Dataset containing 2,715,951 SNPs for 258 unique genets. PCA: PLINK v1.90b6.21 was used to conduct PCA. Since the first PC (PC1) explained 75.57% of the variance, a bar plot was made by ggplot2 package (for R) based on PC1. Neighbor-joining (NJ) tree: pairwise genetic distance was calculated by PLINK (genetic distance index: 1-ibs), which was converted to MEGAX input format (.meg). A NJ tree was then made by MEGAX v10.2.6.

STRUCTURE v2.3.4 analysis: An additional SNP filtering was conducted on the Core Dataset for STRUCTURE analysis. VCFtools was used to keep only SNPs with minor allele frequency (maf) > 0.2, and to thin the dataset by keeping only one SNPs within a 5000 bp window. Among the remaining SNPs, we randomly selected 13,056 SNPs for STRUCTURE analysis (K = 2). Twenty independent runs were conducted, and they all produced consistent results.

Detecting triploidy using Variant read frequency (VRF) histograms

Homologous chromosomes may carry different alleles. Assuming those are bi-allelic loci, diploid individuals would have equal number of reference allele (i.e., REF allele in the vcf file) and variant allele (ALT allele in the vcf file), corresponding to a variant allele frequency of 0.5. This will lead to a peak at 0.5 in the variant read frequency (VRF) histogram (VRF, the number of NGS reads supporting the ALT allele/ total number of NGS reads). Similarly, triploid individuals would have 0, 1, 2, 3 copies of the variant allele, corresponding to a variant allele frequency of 0, 1/3, 2/3, and 1, respectively. Therefore, there would be peaks at 1/3 or 2/3, or both. For each sample, all the

heterozygous genotypes were selected, and the VRF was calculated. Then, a VRF histogram was plotted. This was conducted for all the samples, including the redundant ramets of the same clone.

Detecting F1 hybrids and higher order hybridization

F1 hybrids refer to the direct offspring of two different species or highly diverged clades. Any fixed genetic differences between two divergent taxa would consistently lead to heterozygous genotypes in F1 hybrids. Higher order hybrids such as the offspring of the F1 generation would inevitably lose 50% of the heterozygosity through Mendelian segregation, both in back-crossing with parent species or in F2 hybrids. We first identified the fixed differences between Nj_N and Nj_S, and then checked genotypes of hybrids at those SNPs. For each hybrid sample, the proportion of heterozygous genotypes was calculated, i.e., number of heterozygous SNPs / total number of SNPs with available genotypes. A value equal or very close to 100% would indicate F1 hybrids.

Time-calibrated phylogenetic tree

The *Z. marina* sample used for constructing the reference genome represented a spatially extended and possibly old gene[24,25] containing somatically derived heterozygous genotypes via somatic genetic mutations and drift that could bias the time estimate. Therefore, we decided to sequence two seedlings that will feature only a few somatic mutations, none of which can reach fixation.

Two *Z. marina* seedlings and the unique genets for *N. japonica* and *N. noltii* were used for joint SNP calling and filtering. SNAPP analysis was conducted to build a time-calibrated phylogenetic tree, using the divergence time between *Z. marina* and *N. japonica* as calibration point[23]. Three different sets of samples were used to check the consistency of results, and three independent SNAPP runs were conducted for each set of samples. Only *N. japonica* populations marked with the orange or blue colors in Fig. 2a were included, excluding the admixed populations.

Chloroplast haplotype analysis

The clean NGS reads were mapped to the chloroplast reference genome of *N. japonica*[26]. The process from fastq files to raw SNP dataset was same with the method used for nuclear SNPs. Starting from the raw SNP dataset, BCFtools v 1.15.1 was used to remove SNPs within 20 base pairs of an indel or other variant type, as these variant types may cause erroneous SNPs calls. VCFtools was used to keep SNPs with two different alleles, and the same set of samples removed in the nuclear analyses were also excluded here. VCFtools was used to convert genotypes with coverage > 30 to missing data, and then used to keep only SNPs without any missing data among all samples. Finally, VCFtools was used to keep only polymorphic SNPs (427 SNPs). The allelic state was decided based on the variant read frequency. REF allele and ALT allele were assigned when VRF < 0.65 and VRF > 0.65, respectively. PopART was used to construct a haplotype network.

Karyotyping

We also verified the inferred ploidy level by karyotyping. Since such an analysis required live

plants, it was precluded for already DNA sequenced samples. As triploidy will also be reflected in multi-allelic patterns at polymorphic marker loci, we genotyped both putatively triploid genets at 17 microsatellite loci (19 ramets in total, Supplementary Data Table 1) based on DNA extracts of a piece of a leaf. A diagnostic subset of 9 microsatellite loci[30] was then used to genotype 123 live plant samples from the population YUL with putatively triploid ramets (Supplementary Data Table 2).

Live samples with multiple ramets were collected from the population YUL, and they were transported to Qingdao, China within 24 hours. Each sample was planted into the sediment in one beaker placed in the tank with circulating sea water. Putatively diploid and triploid individuals were identified using microsatellite markers. Then, two putatively diploid samples and one putatively triploid sample were removed from the sediments and cleaned with seawater. The three samples were immediately transported to OMIX Technologies Corporation (Chengdu, China), where they were soaked in the ambient seawater to wait for the formation of new root tips needed for karyotyping.

The root tips with a length of 2-3 cm were pretreated in nitrous oxide for 2h under 1MPa. The root tips containing dividing cells were dissected and digested 2% cellulase Onozuka R-10 and 1% pectolyase Y23 (Yakult Pharmaceutical, Japan) solution for 1h at 37°C.The cells were then collected by centrifugation and resuspended in 90% acetic acid. The droplets from the cell suspension were then placed on glass slides contained in a box lined with wet paper. The fluorescence staining of the chromosomes was performed using 4',6-diamidino-2-phenylindole (DAPI), as previously described[27]. After DAPI staining, the dispersed metaphase chromosome cells were counted under a fluorescence microscope (Zeiss LSM880, Germany). Accurate karyotyping was confirmed by fluorescence in situ hybridization (FISH), as previously described[28]. A telomere-specific-repeat probe (5'-TTTAGGGTT TAGGGTTTAGGG-3') was used to confirm the number of intact chromosomes. The images were captured with an Olympus BX53 fluorescence microscope (OMIX Technologies Corporation).

Comparison of the two chromosome-level reference genomes

To check whether inversions play a role in hybrid sterility, we compared the Nj_N and Nj_S reference genomes. JCVI was used to conduct synteny comparison[29]. GFF format was converted to BED format using jcvi.formats.gff, and fasta files were reformatted using jcvi.formats.fasta. Pairwise synteny comparison was conducted using jcvi.compara.catalog, and visualized with jcvi.graphics.karyotype. Standard sequence alignment was generated by NUCmer (NUCleotide MUMmer) pipeline[30]. To reveal which samples were homozygous or heterozygous for the inversion, a PCA was conducted based on the 131,306 SNPs located in the inversion region.

References

1 Tomlinson, P. B. & Posluzny, U. Generic limits in the seagrass family Zosteraceae. *Taxon* **50**, 429-437 (2001).

2 Sullivan, B. K. & Short, F. T. Taxonomic revisions in Zosteraceae (*Zostera*, *Nanozostera*, *Heterozostera* and *Phyllospadix*). *Aquat. Bot.* **187** (2023).

3 Coyer, J. A. *et al.* Phylogeny and temporal divergence of the seagrass family Zosteraceae using one nuclear and three chloroplast loci. *Systematics and Biodiversity* **11**, 271-284 (2013).

4 Koren, S. *et al.* Canu: scalable and accurate long-read assembly via adaptive k-mer weighting and repeat separation. *Genome. Res.* **27**, 722-736 (2017).

5 Liu, H., Wu, S., Li, A. & Ruan, J. SMARTdenovo: a de novo assembler using long noisy reads. *Gigabyte* **2021** (2021).

6 Walker, B. J. *et al.* Pilon: an integrated tool for comprehensive microbial variant detection and genome assembly improvement. *PLOS. One.* **9**, e112963 (2014).

7 Burton, J. N. *et al.* Chromosome-scale scaffolding of de novo genome assemblies based on chromatin interactions. *Nat. Biotechnol.* **31**, 1119-1125 (2013).

8 Xu, M. *et al.* TGS-GapCloser: a fast and accurate gap closer for large genomes with low coverage of error-prone long reads. *GigaScience* **9**, giaa094 (2020).

9 Garrison, E. & Marth, G. Haplotype-based variant detection from short-read sequencing. *Preprint at https://arxiv.org/abs/1207.3907* (2012).

10 Li, H. Aligning sequence reads, clone sequences and assembly contigs with BWA-MEM. *Preprint at https://arxiv.org/abs/1303.3997* (2013).

11 Li, H. *et al.* The sequence alignment/map format and SAMtools. *Bioinformatics* **25**, 2078-2079 (2009).

12 Manni, M., Berkeley, M. R., Seppey, M., Simão, F. A. & Zdobnov, E. M. BUSCO update: novel and streamlined workflows along with broader and deeper phylogenetic coverage for scoring of eukaryotic, prokaryotic, and viral genomes. *Mol. Biol. Evol.* **38**, 4647-4654 (2021).

13 Ou, S. *et al.* Benchmarking transposable element annotation methods for creation of a streamlined, comprehensive pipeline. *Genome Biology* **20**, 275 (2019).

14 Flynn, J. M. *et al.* RepeatModeler2 for automated genomic discovery of transposable element families. *Proceedings of the National Academy of Sciences* **117**, 9451-9457 (2020).

15 Brůna, T., Hoff, K. J., Lomsadze, A., Stanke, M. & Borodovsky, M. BRAKER2: Automatic Eukaryotic Genome Annotation with GeneMark-EP+ and AUGUSTUS Supported by a Protein Database. *bioRxiv*, 2020.2008.2010.245134 (2020).

16 Brůna, T., Lomsadze, A. & Borodovsky, M. GeneMark-EP+: eukaryotic gene prediction with self-training in the space of genes and proteins. *NAR Genomics and Bioinformatics* **2** (2020).

17 Stanke, M., Diekhans, M., Baertsch, R. & Haussler, D. Using native and syntenically mapped cDNA alignments to improve de novo gene finding. *Bioinformatics* **24**, 637-644 (2008).

18 Grabherr, M. G. *et al.* Full-length transcriptome assembly from RNA-Seq data without a reference genome. *Nature Biotechnology* **29**, 644 (2011).

19 Haas, B. J. *et al.* Automated eukaryotic gene structure annotation using EVidenceModeler and the Program to Assemble Spliced Alignments. *Genome Biology* **9**, R7 (2008).

20 Li, H. Minimap2: pairwise alignment for nucleotide sequences. *Bioinformatics* **34**, 3094-3100 (2018).

21 Shumate, A., Wong, B., Pertea, G. & Pertea, M. Improved transcriptome assembly using a hybrid of long and short reads with StringTie. *PLOS Computational Biology* **18**, e1009730 (2022).

22 Levy Karin, E., Mirdita, M. & Söding, J. MetaEuk—sensitive, high-throughput gene discovery, and annotation for large-scale eukaryotic metagenomics. *Microbiome* **8**, 48 (2020).

23 Yu, L. *et al.* Ocean current patterns drive the worldwide colonization of eelgrass (*Zostera marina*). *Nat. Plants.* **9**, 1207-1220 (2023).

24 Ma, X. *et al.* Improved chromosome-level genome assembly and annotation of the seagrass, *Zostera marina* (eelgrass). *F1000Research* **10**, 289 (2021).

25 Olsen, J. L. *et al.* The genome of the seagrass *Zostera marina* reveals angiosperm adaptation to the sea. *Nature* **530**, 331-335 (2016).

26 Chen, J. *et al.* Comparative chloroplast genomes of Zosteraceae species provide adaptive evolution insights into seagrass. *Front. Plant. Sci.* **12**, 741152 (2021).

27 Kolano, B., Bednara, E. & Weiss-Schneeweiss, H. Isolation and characterization of reverse transcriptase fragments of LTR retrotransposons from the genome of *Chenopodium quinoa* (Amaranthaceae). *Plant. Cell. ReP.* **32**, 1575-1588 (2013).

28 Jiang, J. M. *et al.* Centers with more therapeutic modalities are associated with improved outcomes for patients with hepatocellular carcinoma. *J. Gastrointest. Oncol.* **10**, 546 (2019).

29 Tang, H. *et al.* JCVI: A versatile toolkit for comparative genomics analysis. *iMeta*, e211 (2024).

30 Marçais, G. *et al.* MUMmer4: A fast and versatile genome alignment system. *PLoS Computat Biol* **14**, e1005944 (2018).

31 Ma, X. *et al.* Seagrass genomes reveal ancient polyploidy and adaptations to the marine environment. *Nat. Plants.* **10**, 240-255 (2024).
